# Mineralization of a Fully Halogenated Organic Compound by Persulfate under Conditions Relevant to in Situ Reduction and Oxidation: Reduction of Hexachloroethane by Ethanol Addition Followed by Oxidation

**DOI:** 10.1021/acs.est.3c03489

**Published:** 2023-08-28

**Authors:** Tae-Kyoung Kim, David L. Sedlak

**Affiliations:** Department of Civil and Environmental Engineering University of California, Berkeley, California 94720, United States

**Keywords:** halogenated solvents, reductive dehalogenation, chlorate

## Abstract

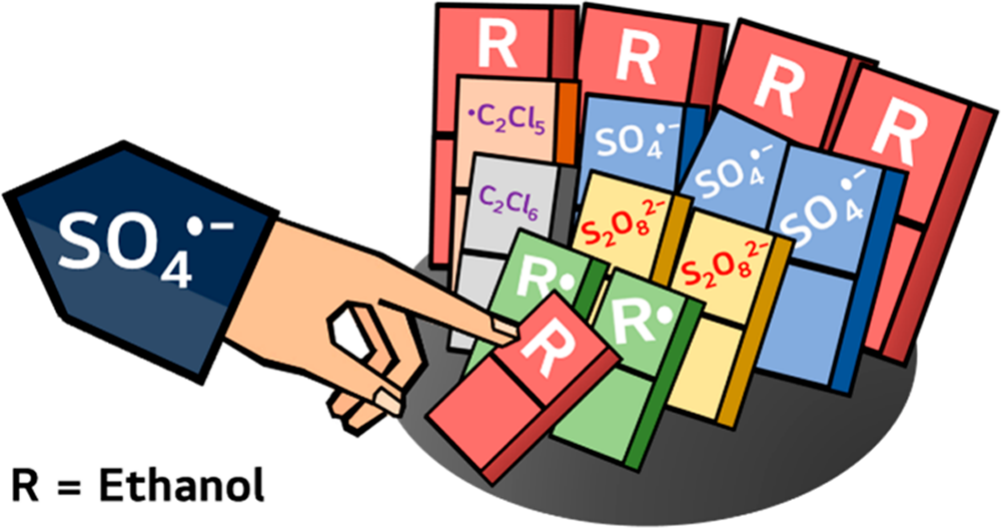

Fully halogenated compounds are difficult to remediate by in situ chemical oxidation (ISCO) because carbon–halogen bonds react very slowly with the species that typically initiate contaminant transformation: sulfate radical (SO_4_^•–^) and hydroxyl radical (^•^OH). To enable the remediation of this class of contaminants by persulfate (S_2_O_8_^2–^)-based ISCO, we employed a two-phase process to dehalogenate and oxidize a representative halogenated compound (i.e., hexachloroethane). In the first phase, a relatively high concentration of ethanol (1.8 M) was added, along with concentrations of S_2_O_8_^2–^ that are typically used for ISCO (i.e., 450 mM). Hexachloroethane underwent rapid dehalogenation when carbon-centered radicals produced by the reaction of ethanol and radicals formed during S_2_O_8_^2–^ decomposition reacted with carbon–halogen bonds. Unlike conventional ISCO treatment, hexachloroethane transformation and S_2_O_8_^2–^ decomposition took place on the time scale of days without external heating or base addition. The presence of O_2_, Cl^–^, and NO_3_^–^ delayed the onset of hexachloroethane transformation when low concentrations of S_2_O_8_^2–^ (10 mM) were used, but these solutes had negligible effects when S_2_O_8_^2–^ was present at concentrations typical of in situ remediation (450 mM). The second phase of the reaction was initiated after most of the ethanol had been depleted when thermolytic S_2_O_8_^2–^ decomposition resulted in production of SO_4_^•–^ that oxidized the partially dehalogenated transformation products. With proper precautions, S_2_O_8_^2–^-based ISCO with ethanol could be a useful remediation technology for sites contaminated with fully halogenated compounds.

## Abstract 2

A new approach is described for in situ remediation of unreactive contaminants by adding ethanol to facilitate reductive dehalogenation.

## Introduction

In situ chemical oxidation (ISCO), the practice of introducing relatively high concentrations of oxidants into groundwater and soil, has proven to be an effective means of rapidly remediating sites that are contaminated with a variety of organic contaminants.^1−3^ Among the various reagents used for ISCO, persulfate (typically added as a solution of 400–1000 mM persulfate (S_2_O_8_^2–^)) has proven to be one of the most effective means of transforming contaminants because the sulfate radical (SO_4_^•–^) and hydroxyl radical (^•^OH) are produced in a controlled manner through aquifer heating or by addition of a strong base to the oxidant solution.^1,4^ Despite the achievements of heat- and base-activated ISCO involving S_2_O_8_^2–^, the efficacy of this technology has diminished significantly in the presence of highly chlorinated contaminants. This limitation arises from the lack of reactivity of saturated compounds that contain multiple carbon–halogen bonds, such as carbon tetrachloride and hexachloroethane, with SO_4_^•–^ and ^•^OH. As a result, their remediation through traditional ISCO methods is challenging.^1,5,6^

Another approach is needed if S_2_O_8_^2–^ is to be used to remediate sites that contain fully halogenated compounds or compounds with multiple difficult-to-oxidize functional groups (e.g., nitro groups). One possible approach involves the use of carbon-centered radicals produced when an oxidizing radical abstracts an electron from a low molecular weight organic compound. For example, Peyton et al.^7^ reported the reduction of carbon tetrachloride and dinitrotoluene when the UV/H_2_O_2_ and UV/ozone processes were employed in the presence of ethanol, respectively. More recently, Zhu et al.^8^ demonstrated the ability of ethanol radicals, produced by heat activation of S_2_O_8_^2–^ under oxygen-free solutions, to dehalogenate carbon tetrachloride.

Despite its ability to reduce organic contaminants, the potential for employing carbon-centered radicals for remediation is uncertain due to the large number of competing reactions that take place when S_2_O_8_^2–^ decomposes in the presence of low molecular weight organics. O_2_, Cl^–^, and HCO_3_^–^ also can decrease the efficiency of the process by competing for carbon-centered radicals, SO_4_^•–^, ^•^OH, or one of the intermediates produced during the decomposition of S_2_O_8_^2–^ (e.g., S_2_O_8_^•–^).^9,10^ Chain reactions can also accelerate the S_2_O_8_^2–^ decomposition rates. For example, the rate of decomposition of S_2_O_8_^2–^ ([S_2_O_8_^2–^]_0_ = 40 mM) in the presence of 0.05 M was 9 × 10^–5^ min^–1^. It increased by a factor of approximately 1.1 (1 × 10^–4^ min^–1^) and 2.2 (2 × 10^–4^ min^–1^) in the presence of 0.2 and 0.4 M ethanol, respectively.^11^ For ethanol, the products of transformation reactions initiated by SO_4_^•–^ or ^•^OH (e.g., acetaldehyde) also can participate in radical reactions, altering the concentrations of radicals involved in dehalogenation or S_2_O_8_^2–^ decomposition reactions. Finally, except for fully halogenated compounds that are essentially unreactive with SO_4_^•–^ and ^•^OH, organic contaminants are likely to undergo dehalogenation and oxidation reactions simultaneously when S_2_O_8_^2–^ decomposes in the presence of ethanol, further complicating efforts to understand the reaction mechanisms.

To provide insight into the potential for using carbon-centered radicals to remediate groundwater that has been contaminated with halogenated compounds that are difficult to oxidize with SO_4_^•–^ or ^•^OH, we evaluated the use of S_2_O_8_^2–^ and ethanol to transform hexachloroethane, a fully halogenated compound that would be impractical to treat by conventional ISCO. To understand conditions likely to be encountered during ISCO treatment, the reactions were studied under acidic conditions (i.e., pH < 2) typical of those encountered when high concentrations of S_2_O_8_^2–^ are employed.^12^ By monitoring concentrations of hexachloroethane, S_2_O_8_^2–^, O_2_, and transformation products under various conditions, we were able to assess reaction mechanisms and the impact of solutes that could interfere with the transformation process. By adding an excess of S_2_O_8_^2–^ relative to the amount of ethanol initially added to the system, we were able to create a second phase of the process in which partially dehalogenated reaction products were further transformed and partially mineralized by SO_4_^•–^ or ^•^OH.

## Materials and Methods

Unless otherwise specified, chemicals were obtained at the highest available purity and used without further purification. A list of chemicals and their sources is summarized in Text S1.

Two sets of stock solutions of 500 mM Na_2_S_2_O_8_ were prepared in deionized water from a Milli-Q ultrapure water system (18.2 MΩ·cm at 25 °C). One set was degassed at room temperature by bubbling with N_2_ to remove O_2_. To adjust the desired [O_2_]_0_, a deoxygenated NaS_2_O_8_ solution was mixed with air-saturated or deoxygenated deionized water and ethanol. The other set was allowed to equilibrate with the atmosphere for at least 30 min prior to use and mixed with the ethanol solution.

To study conditions typical of those employed for remediation, a solution of target compound or ethanol was added to the Na_2_S_2_O_8_ stock solution (i.e., typically 10 μL of target compound solution dissolved in ethanol and 4.99 mL of purged pure ethanol was added to 45 mL of Na_2_S_2_O_8_ stock solution). The initial pH of the solution was around 1.5 due to the partial decomposition of S_2_O_8_^2–^ in the stock solution when 10 mM persulfate solution was prepared.

Experiments were also conducted at a much lower initial concentration of S_2_O_8_^2–^ (i.e., 10 mM). Under these conditions, a small volume of Na_2_S_2_O_8_ stock solution was added to the target compound dissolved in an ethanolic solution (i.e., typically 1 mL of 500 mM Na_2_S_2_O_8_ stock solution was added to 49 mL of target compound solution dissolved in an ethanolic solution; each solvent was separately purged before mixing). Without an added buffer, the initial pH of this solution was around 4.3 under this condition.

After mixing, solutions were transferred to 9 mL amber glass vials without headspace and sealed with a Teflon cap. Capped vials were heated in a water bath at temperatures ranging from 30 to 50 °C. To conduct experiments at 2 °C, vials were placed in a refrigerator. Samples placed in warm water baths reached the target temperature within 5 min; samples placed in the refrigerator required about 3.5 h to reach the target temperature (Figure S1). At predetermined times, samples from the water bath were transferred to an ice bath to stop the reaction. After 2 min of cooling, 1 mL aliquots were transferred to 2 mL GC vials containing 1 mL of *n*-hexane and capped. Liquid–liquid extraction was conducted in the vial on a shaker for 30 min at room temperature prior to the analysis of an aliquot of the hexane layer by a gas chromatography mass spectrometer (GC/MS).

Hexachloroethane, pentachloroethane, tetrachloroethene, and trichloroethene were analyzed by GC/MS (8890 GC and 5977B MS, Agilent, Santa Clara, CA, US). The method detection limit (MDL) for hexachloroethane was 18 nM. MDLs for pentachloroethane, tetrachloroethene, and trichloroethene were 13, 9, and 21 nM, respectively. A modified version of the U.S. EPA 8270D GC/MS analysis method^13^ was used. An example chromatogram and details are included in Text S2 and Figure S2. The calibration result of the halogenated compounds is shown in Figure S3.

Chloride (Cl^–^), sulfate (SO_4_^2–^), and chlorate (ClO_3_^–^) concentrations were determined by a modified version of U.S. EPA 300.1 method^14^ with ion chromatography (Dionex Aquion IC System., ThermoFisher Scientific, Waltham, MA, US), an Ion Pac column (AS23, 4 × 250 mm), and a guard column (AG23, 4 × 50 mm). The column temperature was set to 30 °C, and AS23 eluent (4.5 mM NaHCO_3_ and 0.8 mM Na_2_CO_3_) was used. The eluent flow rate was 1.0 mL min^–1^, and the current was 25 mA. A typical anion calibration curve is shown in Figure S4. Under the highly acidic conditions used in these experiments, nearly all of the carbonate species are converted into carbonic acid species (i.e., H_2_CO_3_^*^) and gaseous carbon dioxide. Including carbonate in the solutions resulted in the presence of gas bubbles that complicate the analysis of volatile products. Carbonic acid is relatively unreactive with SO_4_^•–^ and ^•^OH, especially in the presence of high concentrations of ethanol and other organic solutes. Therefore, we excluded carbonate from these experiments.

Benzoic acid concentrations were determined by HPLC-DAD (G1311B, Agilent, Santa Clara, CA, US) with a reverse-phase column (Hydro-RP, 4 μm, 150 × 3 mm, Phenomenex, Torrence, CA, US). Formic acid (0.1%, v/v) and methanol (60:40 v/v %) were used as the eluent with a 0.4 mL min^–1^ flow rate. UV absorbance was measured at 254 nm. A benzoic acid calibration curve is shown in Figure S5.

S_2_O_8_^2–^ was quantified using a modified version of a previously described method (Text S3).^15,16^ A representative S_2_O_8_^2–^ calibration curve is shown in Figure S6. Dissolved oxygen ([O_2_]) and pH were measured with an Orion STAR A213 meter coupled with the Orion dissolved oxygen probe and a DB-10 pH meter (Denver Instruments, Bohemia, NY, US). To ensure accurate O_2_ analysis, the probe was polished with a polishing disk (Product number: 080513) whenever needed due to corrosion by the high concentration of S_2_O_8_^2–^. Temperature was measured with an alcohol thermometer (ThermoFisher Scientific, Waltham, MA, US).

## Results and Discussion

### Halogenated Contaminant Reduction

**Figure 1:**
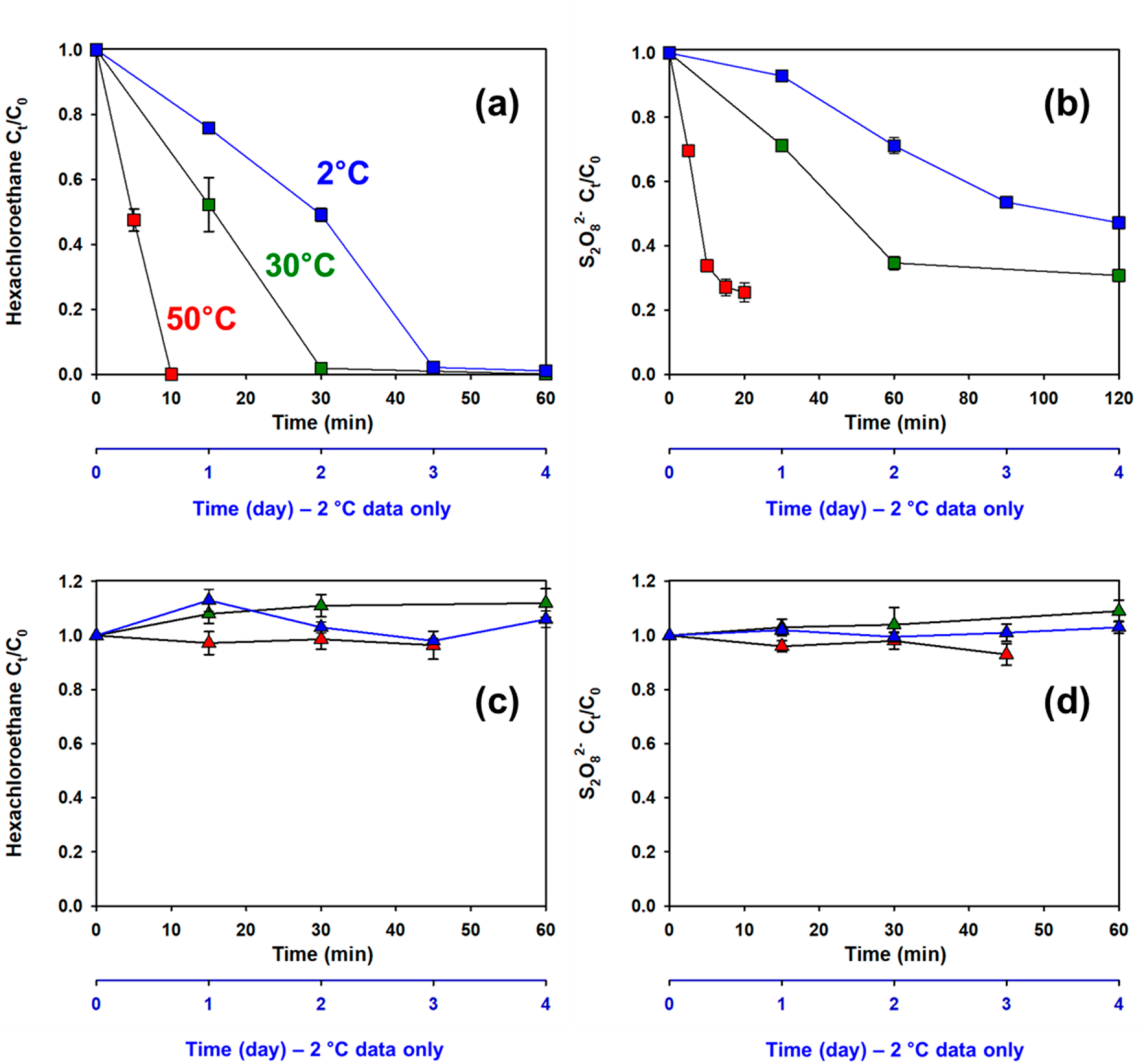
Hexachloroethane transformation (a) and S_2_O_8_^2–^ consumption (b) during simulated ISCO treatment in the presence of ethanol and typical concentrations of groundwater solutes (anions and O_2_) at [S_2_O_8_^2–^]_0_ = 450 mM and at [S_2_O_8_^2–^]_0_ = 10 mM (c, d). *X* axis offset (blue color, day scale) indicates the time scale of 2 °C data. 30 °C and 50 °C data refer to the *X* axis (black color, min scale). Red, green, and blue symbols represent 50 °C, 30 °C, and 2 °C data, respectively. Conditions: [Ethanol]_0_ = 0.18 mM and 1.8 M; [O_2_]_0_ = 220 to 275 μM; [Hexachloroethane]_0_ = 50 μM; [Cl^–^]_0_ = 1 mM; [NO_3_^–^]_0_ = 0.1 mM; [NO_2_^–^]_0_ = 0.01 mM; pH_0_ = 1.4 for [S_2_O_8_^2–^]_0_ = 450 mM; pH_0_ = 4.3 for [S_2_O_8_^2–^]_0_ = 10 mM.

In solutions that initially contained relatively high concentrations of S_2_O_8_^2–^ (i.e., 450 mM) and ethanol (1.8 M) along with solutes typically encountered in groundwater (i.e., O_2_, Cl^–^, NO_2_^–^, and NO_3_^–^), a decrease in the concentration of S_2_O_8_^2–^ coincided with transformation of hexachloroethane. At 50 °C, hexachloroethane concentrations decreased by over 99.96% (i.e., to concentrations below the method detection limit) in about 10 min as [S_2_O_8_^2–^] decreased by about 66%. Similar results were observed at 30 °C, with slightly slower rates of transformation. At 2 °C, complete transformation of hexachloroethane required 3 days and consumed a little less than half of the S_2_O_8_^2–^. In the presence of a lower concentration of S_2_O_8_^2–^ (i.e., 10 mM), no measurable hexachloroethane or S_2_O_8_^2–^ transformation was observed over these time intervals (Figure 1c,d). Less than 1% of the hexachloroethane (Figure S7a) and less than 1.7 mM of the S_2_O_8_^2–^ were transformed over 3 days in control experiments conducted at 2 °C (Figure S7b) in the presence of much lower concentrations of ethanol (i.e., 0.00022 M) with an initial S_2_O_8_^2–^ concentration of 450 mM.

Under conditions that resulted in complete loss of hexachloroethane by S_2_O_8_^2–^ in the presence of ethanol, only 9.4% and 12.8% of the hexachloroethane was transformed when ethanol was replaced by either *tert*-butanol or methanol, respectively (Figure S8a). The faster rate of hexachloroethane transformation in the presence of ethanol was accompanied by greater S_2_O_8_^2–^ loss; after 30 min, more than 60% of S_2_O_8_^2–^ decomposition was observed in the presence of ethanol, whereas 2.7% and 4.8% of S_2_O_8_^2–^ had disappeared in the presence of *tert*-butanol and methanol, respectively (Figure S8b). These findings are consistent with results from a previous study^11^ in which faster S_2_O_8_^2–^ decomposition rates in the presence of high concentrations of ethanol were explained by differences in the abilities of alcohols and certain alcohol transformation products, typically aldehydes, to terminate radical chain reactions.

The rate of disappearance of S_2_O_8_^2–^ in the presence of 1.8 M ethanol (Figure 1b) was substantially faster than expectations based on measurements of its rate of thermolysis (reaction 1) in pure water (i.e., predicted half-lives for S_2_O_8_^2–^ thermolysis ranged from 3 days at 50 °C to 14 years at 2 °C).^17^ The faster-than-expected decay of S_2_O_8_^2–^ may be attributable to radical chain reactions initiated by the reaction of SO_4_^•–^ and ethanol (reactions 2 to 3),^17,18^ and the decomposition rate constants of S_2_O_8_^2–^ can be determined by the sum of the thermal activation (reaction 1).^19,20^ (All rate constants in the text were measured at temperatures ranging from 20 to 25 °C as described in the cited references.)
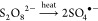
1See ref (18):
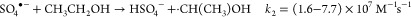
2
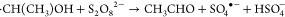
3Acetaldehyde (i.e., CH_3_CHO), produced in reaction 3 undergoes a similar set of reactions (reactions 4 and 5):^11^See ref (18):
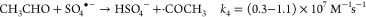
4
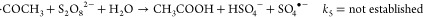
5The carbon centered radical produced in reaction 4 (i.e., ·COCH_3_) tends to be less reactive with organic compounds and S_2_O_8_^2–^ than the ethanol radical as indicated by the slower rate of hexachloroethane transformation (Figure S9a) and S_2_O_8_^2–^ decomposition observed when ethanol was replaced by acetaldehyde (Figure S9b).

**Figure 2:**
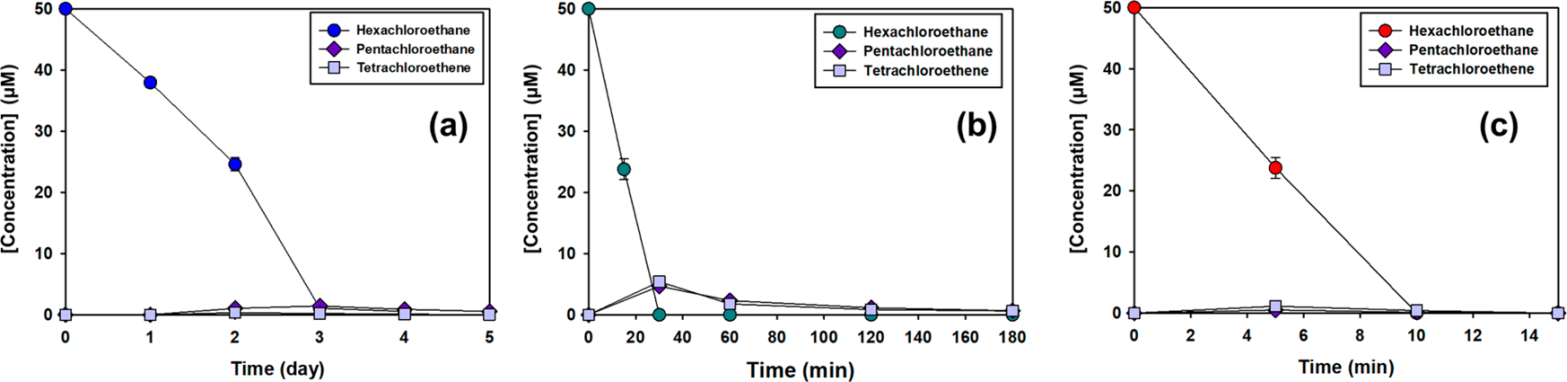
Loss of hexachloroethane and formation of dehalogenated products at 2 °C (a); 30 °C (b); 50 °C (c) ([Ethanol]_0_ = 1.8 M, [O_2_]_0_ = 275 μM, [S_2_O_8_^2–^]_0_ = 450 mM, [Cl^–^]_0_ = 1 mM, [NO_3_^–^]_0_ = 0.1 mM, [NO_2_^–^]_0_ = 0.01 mM, pH_0_ = 1.4).

The transformation of hexachloroethane is likely due to hydrogen abstraction (reactions 6 and 7)^21,22^ or pentachloroethane carbanion protonation (reactions 8 and 9):^21−23^
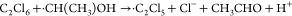
6
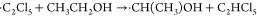
7
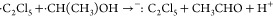
8
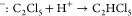
9In agreement with this mechanism,^21−24^ low concentrations of pentachloroethane, typically less than 1% of the amount of hexachloroethane that was transformed, were detected during transformation of hexachloroethane at all three temperatures tested (Figures 2 and S11).

The pentachloroethyl radical and pentachloroethane carbanion also can produce tetrachloroethene by a reaction with ethanol radical (reaction 10)^20,22,24^ or dechlorination of pentachloroethyl carbanion via an internal two electron transfer (reaction 11):^20,22,24^
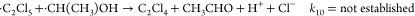
10
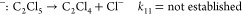
11In accordance with these mechanisms, tetrachloroethene formation was observed under all reaction conditions (Figures 2 and S11).

The relative concentrations of pentachloroethane and tetrachloroethene formed during the transformation of hexachloroethane varied with temperature, with relative concentrations of tetrachloroethene increasing with temperature (Figures 2 and S10). Changes in the relative concentrations of transformation products with temperature may have been caused by differences in the activation energies of the various reactions responsible for the formation of each of the measured products as well as subsequent reactions that lowered the concentrations of measured transformation products.

To investigate the mass balance and chloride recovery during hexachloroethane transformation, experiments were conducted at a lower [S_2_O_8_^2–^]_0_ (i.e., 10 mM). Under these conditions, pentachloroethane and tetrachloroethene accounted for less than 40% of the carbon loss at the end of the experiment (Figure S11a,c,e), while measured [Cl^–^] was 50–100% higher than values predicted if pentachloroethane and tetrachloroethene were the only transformation products (Figure S11b,d,f).

Although pentachoroethane can undergo an E_2_ reaction to produce tetrachloroethene,^22,25^ we ruled out the importance of this reaction under the conditions employed here through control experiments in which pentachloroethane was exposed to S_2_O_8_^2–^ and ethanol under pH conditions similar to those used in these experiments (Figure S12).

Despite being able to confirm that the enhanced S_2_O_8_^2–^ decomposition in the presence of ethanol led to Cl^–^ release, further analysis of the intermediates and products is needed to validate the proposed mechanism. Quantification of the steady-state concentration of the carbon-centered radicals, key intermediates, involved in the reductive phase was unfeasible due to the absence of suitable radical probes. For a comprehensive and fundamental understanding of this reaction, additional research will be needed.

### Effect of Dissolved Solutes on Rates of Hexachloroethane Loss

O_2_ competes with hexachloroethane for carbon-centered radicals (reactions 12 and 13):^11,26^See ref (21):
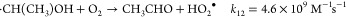
12
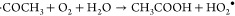
13Hydroperoxyl radical (HO_2_^•^) and its conjugate base, superoxide radical (O_2_^•–^), produced in these reactions rapidly undergo bimolecular dismutation to produce H_2_O_2_ under acidic conditions (reactions 14 and 15):^27^See refs (28 and 29):
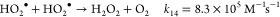
14See refs (28 and 29):
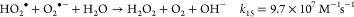
15In the presence of soil or aquifer solids, this conversion would be even faster due to the presence of transition metals that can catalyze (i.e., Fe, Mn, and Cu) the dismutation reaction.^30,31^

**Figure 3:**
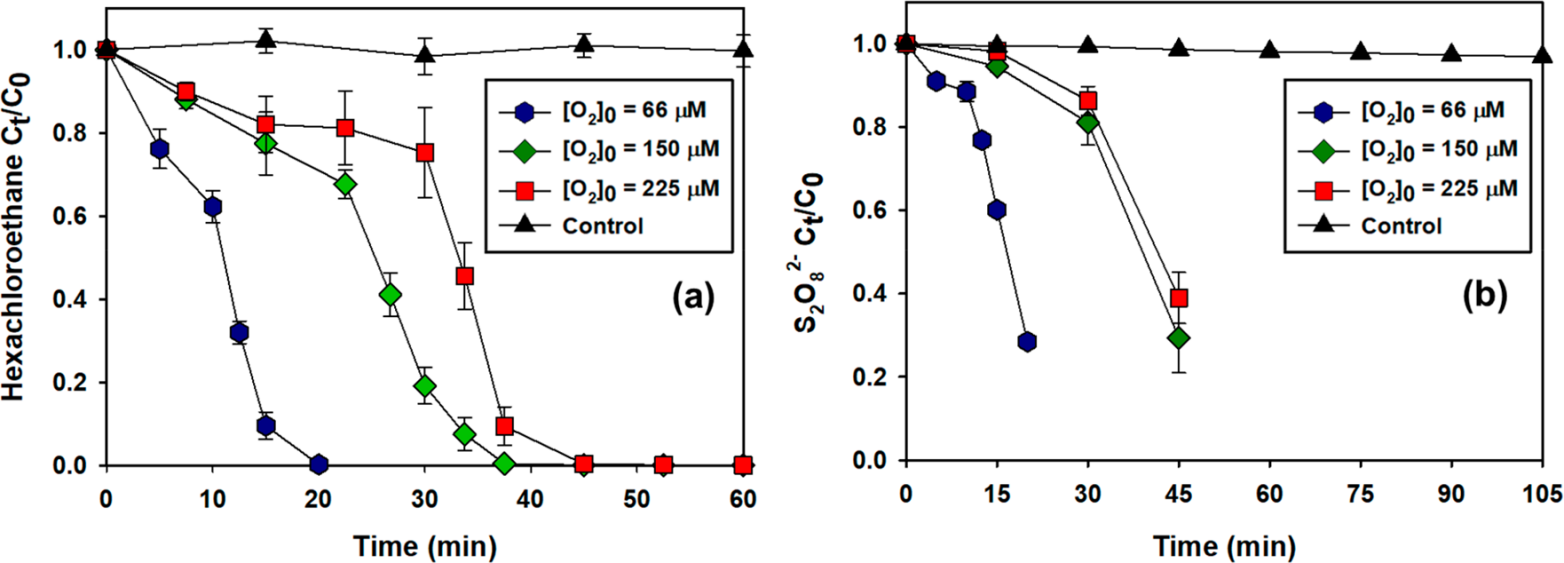
Transformation of hexachloroethane (a) and persulfate decomposition (b) at [S_2_O_8_^2–^]_0_ = 10 mM and varying [O_2_]_0_. Control experiments were conducted at [O_2_]_0_ = 225 μM in the presence of 175 μM ethanol with 10 mM S_2_O_8_^2–^. For other experiments: [Ethanol]_0_ = 1.8 M; *T* = 50 °C; pH_0_ = 4.3.

Due to these competing processes (i.e., reactions 13 and 14), the presence of O_2_ slows the rate of transformation of hexachloroethane as well as the initial loss of S_2_O_8_^2–^ at low initial concentrations of S_2_O_8_^2–^ (i.e., 10 mM; Figure 3). After O_2_ is depleted (Figure S13), the rate of hexachloroethane transformation and S_2_O_8_^2–^ returns to values similar to those observed when O_2_ had been removed prior to initiation of the reaction. When the initial concentration of persulfate was increased to values in the range employed for ISCO treatment (i.e., 450 mM), an initial lag in hexachloroethane removal was not observed for water that was initially saturated with air (i.e., [O_2_]_0_ = 225 μM; Figure 1a). Thus, the presence of O_2_ in solution used during remediation will not have a noticeable effect on the efficacy of the reduction process. It is also unlikely that diffusion of O_2_ into the solution from interstitial air in the vadose zone or reactions that produce O_2_ will affect the kinetics of organic contaminant reduction or rates of S_2_O_8_^2–^ disappearance.

In addition to O_2_, one of the groundwater solutes that is typically present at the highest concentrations, Cl^–^, can be oxidized by SO_4_^•–^ (reaction 16)^18,32^ to form Cl·:See ref (18):
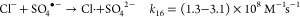
16Cl· may undergo subsequent reactions with Cl^–^ and SO_4_^•–^ to produce ClO_3_^–^.^33,34^ Groundwater contaminants, such as NO_3_^–^ and NO_2_^–^, also can be reduced by species such as solvated electron (e_aq_^•–^) (reactions 17 and 18) or oxidized by SO_4_^•–^ (reaction 19):See ref (18):
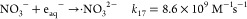
17See ref (18):
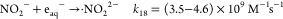
18See ref (18):
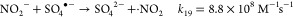
19

To assess the effect of these solutes on hexachloroethane reduction rates, we repeated several of the previously described experiments with environmentally realistic concentrations of each of the solutes and high and low concentrations of [S_2_O_8_^2–^]_0_ in air-saturated solutions containing 1.8 M ethanol. At the relatively high concentrations typical of ISCO treatment (i.e., [S_2_O_8_^2–^] = 450 mM), hexachloroethane reduction was observed even in the presence of anions (Figure 1a). However, at low initial persulfate concentrations ([S_2_O_8_^2–^] = 10 mM), each solute inhibited the transformation of hexachloroethane (Figure S14a) and the conversion of S_2_O_8_^2–^ (Figure S14b). The rate constants for the reactions of ·CH(CH_3_)OH with NO_3_^–^ and NO_2_^–^ are unknown. In addition to the data in Figure S14, these oxidized nitrogen species are also known to react with the strong reductant, solvated electron (reactions 17 and 18). Despite their likely reactivity with ·CH(CH_3_)OH, it appears that these solutes did not depress the steady-state concentration of the radical enough to slow the rate of contaminant transformation at [S_2_O_8_^2–^] = 450 mM. Nevertheless, our initial findings suggest that, under conditions typical of most contaminated sites, solutes present in groundwater are unlikely to appreciably slow transformation. This is because the injection fluid is typically prepared using water sources such as tap water or relatively clean groundwater, which tend to have relatively low concentrations of chloride, nitrogen species, and other solutes that might interfere with the process.

Recognizing that injection fluids could become contaminated by solutes such as Cl^–^ as injected water mixes with native groundwater or that anions may desorb from mineral surfaces exposed to high concentrations of SO_4_^2–^ and S_2_O_8_^2–^, we tested the impact of elevated chloride concentrations on hexachloroethane reduction with high concentrations of S_2_O_8_^2–^ (Figure S15). Under these conditions, the onset of hexachloroethane transformation was delayed by about 2.5–5.0 min at [Cl^–^] ranging from 1 to 50 mM, but complete removal of the compound still occurred within 10 min of initiating the experiment.

To investigate the effect of organic cocontaminants, sinks for ^•^OH and SO_4_^•–^,^36,37^ on the reductive process, an experiment was conducted with low concentrations of S_2_O_8_^2–^ in the presence and absence of the SO_4_^•–^ scavenger, benzoic acid (reaction 20):See ref (35):
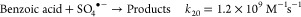
20The reaction rate constant of benzoic acid with SO_4_^•–^ is about 2 orders of magnitude higher than that of ethanol and SO_4_^•–^. Under the conditions studied (i.e., initial benzoic acid concentration 2 orders of magnitude lower than that of ethanol), the benzoic acid should have competed with ethanol for SO_4_^•–^. As a result, the rate of transformation of hexachloroethane decreased by about 50%, relative to the rate observed in the absence of benzoic acid (Figure S16a). The rate of loss of S_2_O_8_^2–^ also slowed in the presence of benzoic acid, presumably due to less initiation of chain decomposition reactions (Figure S16b). Although the presence of a cocontaminant that reacts with SO_4_^•–^ slowed the rate of hexachloroethane reduction at low [S_2_O_8_^2–^]_0_ (i.e., 10 mM), it did not have a measurable effect at higher [S_2_O_8_^2–^]_0_; complete loss of hexachlorobenzene was observed when 450 mM S_2_O_8_^2–^ was added (Figure S17a). Also, under the same condition, there was no significant difference in S_2_O_8_^2–^ decomposition rates in the presence (6.7 × 10^–2^ min^–1^) and absence (7.2 × 10^–2^ min^–1^) of benzoic acid (Figure S17b). We also do not expect inhibition from natural organic matter because it is usually present at relatively low concentrations compared to organic cocontaminants (e.g., typical concentrations of dissolved organic carbon in groundwater are around 1 mg L^–1^).^38^

### Oxidative Compound Removal and Halogenated Byproduct Mineralization

The second phase of this process (oxidation) may be necessary after the fully halogenated compound is lost to transform the dehalogenated transformation products (i.e., the compounds that remained after carbon–halogen bond reduction by carbon-centered radicals; Figure S11 and Text S4). These compounds should be susceptible to oxidation by SO_4_^•–^.

**Figure 4:**
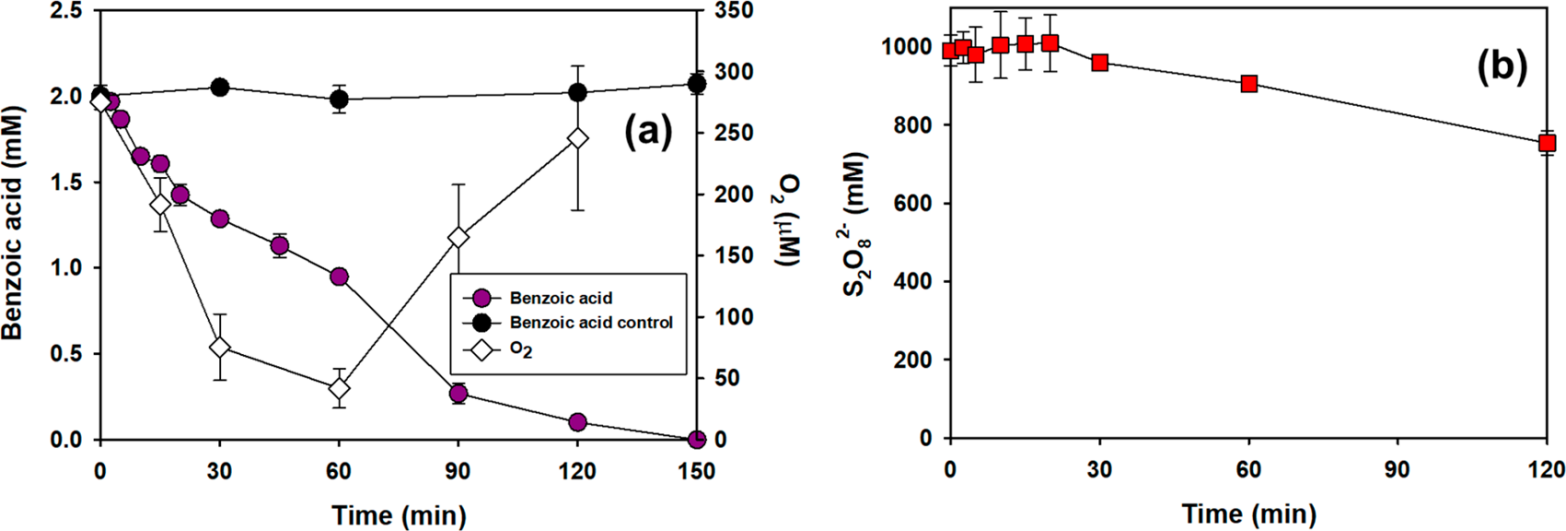
Transformation of [Benzoic acid] (a) and S_2_O_8_^2–^ decomposition (b) during a two-phase experiment. [O_2_] decreased during the first phase (the reduction step) (a). During the second phase (the oxidation step), [O_2_] increased as the rate of the benzoic acid transformation accelerated (a). Conditions: [S_2_O_8_^2–^]_0_ = 990 mM; [Ethanol]_0_ = 175 mM; [Benzoic acid] = 2 mM; Temp = 50 °C, pH_0_ = 1.4, *n* = 2. A control experiment to assess the possibility of volatilization of benzoic acid under acidic pH conditions was conducted using concentrated sulfuric acid, which was added instead of Na_2_S_2_O_8_.

A shift in the mode of action of S_2_O_8_^2–^ should take place as the ethanol, acetaldehyde, and other compounds are mineralized: When the compounds are present at relatively high concentrations (i.e., >100 mM), oxidizable contaminant (i.e., transformation products of fully halogenated compounds) removal should mainly take place through reactions with SO_4_^•–^; the fraction of SO_4_^•–^ that produces reducing radicals will drop, and [SO_4_^•–^]_ss_ will increase. To gain insight into the shift from reducing to oxidizing conditions, we conducted experiments with excess S_2_O_8_^2–^ relative to ethanol, using benzoic acid as a probe for SO_4_^•–^ (Figure 4). During the first 60 min, O_2_ was gradually depleted through reactions with ethanol and its oxidation products (e.g., reactions 12 and 13). During this phase, benzoic acid was slowly oxidized. After 60 min, we observed an increase in the rate of benzoic acid transformation (Figure S18), coinciding with the rise in O_2_ levels due to the oxidation of water by SO_4_^•–^ through a radical chain reaction (net reaction 21):^40^
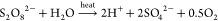
21

During the second phase, partially dehalogenated compounds (e.g., pentachloroethane and tetrachloroethene) are likely to be oxidized, because reductive ethanol radical (*E*^ο^ = −1.25 V vs NHE),^41^ which is present at lower concentrations, is less reactive with these transformation products (Figure S19) when it is compared to oxidative SO_4_^•–^ (*E*^ο^ = 2.5–3.1 V vs NHE).^18^ To simulate the transformation of partially dehalogenated products during the second phase of the process, experiments were conducted in the presence of persulfate at low concentrations of ethanol (i.e., 0.22 mM). Under these conditions, complete mineralization of the unsaturated chlorinated compounds was observed with nearly quantitative recovery of Cl^–^ (Figure S20), which is consistent with previous research on tetrachloroethene oxidation.^42^

## Implications for Groundwater Remediation

The sequential reductive and oxidative treatment process described here has the potential to extend the benefits of in situ chemical remediation with S_2_O_8_^2–^ to fully and partially halogenated compounds that have proven to be difficult to remove by conventional ISCO. In the first phase, SO_4_^•–^ produced from the slow decomposition of S_2_O_8_^2–^ that takes place over a wide range of temperatures produces carbon-centered radicals. These species rapidly reduce halogenated contaminants while simultaneously initiating chain reactions that produce more carbon-centered radicals. The relatively high concentrations of S_2_O_8_^2–^ employed during ISCO overwhelm the inhibitory effects of solutes (e.g., O_2_, Cl^–^, organic cocontaminants) that reduce the efficacy of radical reactions under less extreme conditions. The reactions of carbon-centered radicals result in the formation of partially dehalogenated products that are less amenable to further reduction. If a sufficiently high concentration of S_2_O_8_^2–^ is initially added, the process can enter a second phase in which SO_4_^•–^ produced by S_2_O_8_^2–^ activation oxidizes partially dehalogenated compounds and other organic cocontaminants (e.g., hydrocarbons, aromatic compounds). The second phase of the reaction will require a means of accelerating the rate of S_2_O_8_^2–^ activation (e.g., aquifer heating and base addition).

The fluids injected into the subsurface contain relatively high concentrations of alcohol (i.e., about 5–10% alcohol by volume) and oxidant (i.e., 80–200 g/L NaS_2_O_8_). In addition to the expense and need for precautions in materials handling (e.g., fire hazard), decomposition of S_2_O_8_^2–^ will subject soils and aquifer sediments to extreme conditions (e.g., pH values below 2, high ionic strength, and anion concentrations). Ethanol and its oxidation products are less of a concern because they are oxidized during the second phase of the process. Any residual organics that are not mineralized will likely be degraded by microbes in the soil or groundwater. Nonetheless, exposure to these extreme conditions could lead to metal mobilization, alter soil structure, and impact microbial communities. In addition to research to assess the role of iron and other transition metals that might be released from aquifer solids on the kinetics of reductive processes responsible for contaminant removal, research involving authentic aquifer solids is needed to assess the impacts of this treatment on the physical, chemical, and biological conditions of the subsurface.

## Supporting Information Available

Additional experimental details; temperature vs time profiles; GC/MS chromatogram; calibration curves; transformation of hexachloroethane and S_2_O_8_^2–^ decomposition; enlarged version of hexachloroethane transformation and formation of dehalogenated products; loss of hexachloroethane and formation of dehalogenated products; transformation of pentachloroethane; [O_2_] time profile; effects of anions, initial Cl^–^ concentrations, and benzoic acid on hexachloroethane transformation and S_2_O_8_^2–^ consumption; effects of benzoic acid on hexachloroethane transformation in the presence of high or low concentrations of S_2_O_8_^2–^; degradation kinetics of benzoic acid; transformation of hexachloroethane, pentachloroethane, tetrachloroethene, and trichloroethene and recovery of Cl^–^; pentachloroethane and tetrachloroethene mineralization (PDF)
