# Supplementary material for: Mineralization of a Fully Halogenated Organic Compound by Persulfate under Conditions Relevant to in Situ Reduction and Oxidation: Reduction of Hexachloroethane by Ethanol Addition Followed by Oxidation
Source: Environ Sci Technol. 2023 Aug 28;57(36):13691–8. doi: 10.1021/acs.est.3c03489 (PMC10501115; doi:10.1021/acs.est.3c03489)
Supplement: Supplementary file 1 — es3c03489_si_001.pdf [file es3c03489_si_001.pdf]

Supporting Information for:

Mineralization of a Fully Halogenated Organic Compound by Persulfate: in situ Reduction and Oxidation of Hexachloroethane by Ethanol Addition

Tae-Kyoung Kim and David L. Sedlak*

Department of Civil and Environmental Engineering

University of California, Berkeley

California 94720, United States

Submitted to:

Environmental Science & Technology

April 2023

Texts: 4

Figures: 20

Corresponding Author

David L. Sedlak

Email: sedlak@berkeley.edu

Table of contents

Text S1. Chemicals.
Text S2. Analytical conditions of GC/MS for target compounds analysis.
Text S3. Analytical procedure of $S_2O_8^{2-}$
Text S4. The necessity of second phase of reaction (post-oxidation step).
Figure S1. Temperature versus time profiles in the water bath and refrigerator. The water bath temperatures were set to 30 to 70°C and refrigerator was set to 2°C. The blue symbols and x axis offset indicate the time profile of sample temperature decrease in the refrigerator.
Figure S2. Representative GC/MS chromatogram of 0.76 μM trichloroethene (a), 0.6 μM tetrachloroethene (b), 0.49 μM pentachloroethane (c), and 0.42 μM hexachloroethane (d).
Figure S3. Target halogenated compound calibration curves.
Figure S4. Representative anion calibration curves.
Figure S5. Representative benzoic acid calibration curves.
Figure S6. Representative $S_2O_8^{2-}$ calibration curves.
Figure S7. Transformation of hexachloroethane (a) and $S_2O_8^{2-}$ decomposition (b) in the presence of low concentrations of ethanol. X axis offset (blue color – day scale) indicates the time scale of 2°C data. 30°C and 50°C data refer to X axis (black color – min scale) ($[Ethanol]_0$ = 175 μM, $[O_2]_0$ = 220 to 275 μM, $[S_2O_8^{2-}]_0$ = 450 mM, $[Hexachloroethane]_0$ = 50 μM, $[Cl^-]_0$ = 1 mM, $[NO_3^-]_0$ = 0.1 mM, $[NO_2^-]_0$ = 0.01 mM, T = 2 to 50°C, pH_0 = 1.4).
Figure S8. Hexachloroethane transformation (a) and decomposition of $S_2O_8^{2-}$ during heat-activated persulfate in the presence various alcohols (b) ($[Tertiary\ butanol]_0$ = 2.2 M, $[Methanol]_0$ = 2.2 M, $[Ethanol]_0$ = 1.8 M, $[O_2]_0$ = 150 μM, $[S_2O_8^{2-}]_0$ = 10 mM, $[Hexachloroethane]_0$ = 50 μM, Temp = 50°C, pH_0 = 4.3).
Figure S9. Transformation of hexachloroethane (a) and $S_2O_8^{2-}$ decomposition in the presence of ethanol, acetaldehyde and acetic acid ($[Acetic\ acid]_0$ = 2.2 M, $[Acetaldehyde]_0$ = 2.2 M, $[Ethanol]_0$ = 1.8 M, $[O_2]_0$ = 150 μM, $[S_2O_8^{2-}]_0$ = 10 mM, $[Hexachloroethane]_0$ = 50 μM, Temp = 50°C, pH_0 = 4.3).
Figure S10. Enlarged version of hexachloroethane transformation and formation of dehalogenated products at 2°C (a); 30°C (b); and 50°C (c). ($[Ethanol]_0$ = 1.8 M, $[O_2]_0$ = 275 μM, $[S_2O_8^{2-}]_0$ = 450 mM, $[Cl^-]_0$ = 1 mM, $[NO_3^-]_0$ = 0.1 mM, $[NO_2^-]_0$ = 0.01 mM, pH_0 = 1.4).
Figure S11. Loss of hexachloroethane and formation of dehalogenated products during decomposition of persulfate in the presence of ethanol at initial O_2 concentration ($[O_2]_0$) of 66 μM (a), 150 μM (c), and 225 μM (e). Release of chloride and estimated Cl^- contribution from measured dehalogenated products at $[O_2]_0$ of 66 μM (b), 150 μM (d), and 225 μM (f). Conditions: $[Ethanol]_0$ = 1.8 M, $[S_2O_8^{2-}]_0$ = 10 mM, $[Hexachloroethane]_0$ = 50 μM, Temp = 50°C, pH_0 = 4.3. Total mass balance is a sum of $[hexachloroethane]_t$, $[pentachloroethane]_t$, and $[tetrachloroethene]_t$. Calculated Cl^- can be determined by using following equation: Cl^- calculated = $[pentachloroethane]_t$ + $2[tetrachloroethene]_t$.
Figure S12. Transformation of pentachloroethane in the presence of ethanol without $S_2O_8^{2-}$ and in the presence of $S_2O_8^{2-}$ with 175 uM of ethanol. When persulfate was not added, a few drops of concentrated H_2SO_4 was used to adjust the initial pH value. To prevent oxidation of pnetachloroethane via $SO_4^{\bullet-}$ attack, 1 mM of benzoic acid was applied with $S_2O_8^{2-}$. Solutions were purged by N_2 to remove dissolved O_2. ($[Ethanol]_0$ = 175 μM and 1.8 M, $[O_2]_0$ = 70 μM, $[S_2O_8^{2-}]_0$ = 0 and 10 mM, $[benzoic\ acid]_0$ = 1 mM, $[pentachloroethane]_0$ = 50 μM, Temp = 50°C, pH_0 = 4.3).
Figure S13. $[O_2]$ time profile during hexachloroethane transformation ($[O_2]_0$ = 66 to 225 μM, $[Ethanol]_0$ = 1.8 M, $[S_2O_8^{2-}]_0$ = 10 mM, [Hexachloroethane] = 50 μM, Temp = 50°C, pH_0 = 4.3).

Figure S14. Effects of anions on hexachloroethane transformation (a) and $S_2O_8^{2-}$ consumption (b) under the low persulfate conditions ([Ethanol]$_0$ = 1.8 M, [O_2]$_0$ = 275 μM, [$S_2O_8^{2-}$]$_0$ = 10 mM, [Hexachloroethane] = 50 μM, [Cl^-]$_0$ = 1 mM, [NO_3^-]$_0$ = 0.1 mM, [NO_2^-]$_0$ = 0.01 mM, Temp = 50°C, pH$_0$ = 4.3).
Figure S15. Effect of initial Cl^- concentrations on hexachloroethane transformation (a) and $S_2O_8^{2-}$ consumption (b) under the realistic ISCO conditions ([Ethanol]$_0$ = 1.8 M, [O_2]$_0$ = 275 μM, [$S_2O_8^{2-}$]$_0$ = 450 mM, [Hexachloroethane] = 50 μM, [Cl^-]$_0$ = 1 mM, [NO_3^-]$_0$ = 0.1 mM, [NO_2^-]$_0$ = 0.01 mM, Temp = 50°C, pH$_0$ = 1.4).
Figure S16. Effects of benzoic acid on hexachloroethane transformation (a) and $S_2O_8^{2-}$ consumption (b). Control experiments were conducted in the presence of 175 μM ethanol. ([O_2]$_0$ = 150 μM, [Ethanol]$_0$ = 175 μM and 1.8 M, [$S_2O_8^{2-}$]$_0$ = 10 mM, [Benzoic acid]$_0$ = 2 mM, [Hexachloroethane]$_0$ = 50 μM, Temp = 50°C, pH$_0$ = 4.3).
Figure S17. Effects of benzoic acid on hexachloroethane transformation in the presence of high or low concentrations of $S_2O_8^{2-}$ (a). $S_2O_8^{2-}$ consumption ([$S_2O_8^{2-}$]$_0$ = 450 mM) in the presence and absence of benzoic acid (b). When 10 mM of $S_2O_8^{2-}$ was applied, anions were not added. ([Ethanol]$_0$ = 1.8 M, [O_2]$_0$ = 255 μM, [$S_2O_8^{2-}$]$_0$ = 10 and 450 mM, [Hexachloroethane]$_0$ = 50 μM, [Benzoic acid]$_0$ = 2mM, [Cl^-]$_0$ = 0 and 1 mM, [NO_3^-]$_0$ = 0 and 0.1 mM, [NO_2^-]$_0$ = 0 and 0.01 mM, T = 50°C, pH$_0$ = 1.4 when 450 mM of $S_2O_8^{2}$- was applied, pH$_0$ = 4.3 when 10 mM of $S_2O_8^{2}$- was applied).
Figure S18. Degradation kinetics of benzoic acid during the first and second phases of the sequential treatment process ([Ethanol]$_0$ = 175 mM, [Benzoic acid] = 2 mM, [O_2]$_0$ = 280 μM, [$S_2O_8^{2-}$]$_0$ = 990 mM, Temp = 50°C, pH$_0$ = 1.4, n = 2).
Figure S19. Transformation of hexachloroethane (a); pentachloroethane (b); tetrachloroethene (c); and trichloroethene (d) and recovery of Cl^- ([Hexachloroethane]$_0$ = 50 μM, [Pentachloroethane]$_0$ = 50 μM, [Tetrachloroethene]$_0$ = 50 μM, [Trichloroethene]$_0$ = 50 μM, [O_2]$_0$ = 150 μM, [Ethanol]$_0$ = 1.8 M, [$S_2O_8^{2-}$]$_0$ = 10 mM, T = 50°C, pH$_0$ = 4.2 to 4.5).
Figure S20. Pentachloroethane and tetrachloroethene mineralization during simulated second phase (oxidation) of process ([O_2]$_0$ = 225 μM, [Ethanol]$_0$ = 175 μM, [$S_2O_8^{2}$]$_0$ = 10 mM, [Pentachloroethane]$_0$ = 50 μM, [Tetrachloroethene]$_0$ = 50 μM, Temp = 50°C, pH$_0$ = 4.8). When persulfate was not added, a few drops of concentrated H_2SO_4 was used to adjust the initial pH value. Because chlorinated compounds are volatile, chloride analysis was conducted instead of using DOC to confirm the mineralization of each compound.

Text S1. Chemicals

Hexachloroethane (C_2Cl_6), pentachloroethane (C_2Cl_5H) (> 96%), tetrachloroethylene (C_2Cl_4), trichloroethylene (C_2Cl_3H), benzoic acid ($C_7H_6O_2$), sodium persulfate (NaS_2O_8), sodium chloride (NaCl), sodium nitrite ($NaNO_2$), sodium nitrate ($NaNO_3$), formic acid (FA; CH_2O_2), sodium bicarbonate ($NaHCO_3$), potassium iodide (KI), and potassium hydrogen phthalate ($C_8H_5KO_4$) were purchased from Sigma-Aldrich (St. Louis, MO, US). HPLC grade n-hexane, 200 proof Ethanol (100%; biomolecular grade), AS23 eluent concentrate and Dionex™ Combined Seven Anion Standard II were purchased from ThermoFisher Scientific (Waltham, MA, US). Purchased chemicals were analytical grade (> 99%) except for reagent-grade pentachloroethane (> 96%). Purchased chemicals were used without further purification. The stock solutions were prepared in deionized water obtained from a Milli Q-system (R = 18.2 MΩ/cm, Merck Millipore, Burlington, MA, US) or in ethanol.

Text S2. Analytical conditions of GC/MS for target compounds analysis.

For the halogenated organic compounds separation, the DB-5MS-UI column (15 m x 0.25 mm x 0.25 μm) was used. Helium was used as a carrier gas (1.3 mL min^{-1}). The needle depth and the injection volume were 17 mm and 2 μL, respectively. The needle was cleaned with n-hexane between injections. The injector temperature was set at 250°C. The initial oven temperature was 30°C for 2 min. The oven temperature was increased at a rate of 10°C min^{-1} up to 110°C (8 min). Mass spectrometry source and quadrupole temperature were set at 250°C and 150°C.

Text S3. Analytical procedure of $S_2O_8^{2-}$

The $S_2O_8^{2-}$-containing solutions were diluted with deionized (DI) water to achieve a final concentration of 1 mM or lower. Figure S4 shows the calibration curve of $S_2O_8^{2-}$. Subsequently, 0.1 mL of the diluted samples was transferred to conical tubes containing 4.9 mL of a solution of KI and $NaHCO_3$ ([KI]=0.55 M and [$NaHCO_3$] = 76 mM). The prepared samples were then stored in a dark room for 25 minutes before conducting Ultraviolet-visible spectroscopy analysis at a wavelength of 352 nm.

Text S4. The necessity of a second phase (oxidation of partially dehalogenated products).

$S_2O_8^{2-}$ decomposition slowed after 15 min and 60 min of reaction when the temperatures were maintained at 30°C and 50°C (Figure 1b). Similar trend was reported when acetaldehyde was added to heat-persulfate activation in the presence of ethanol.[1] It indicated that reductive radical chain reaction slowed down mainly due to the acetaldehyde accumulation. Thus, additional $S_2O_8^{2-}$ and heat has to be applied at this point to shift the process into oxidation by oxidizing acuumulated acetaldehyde if target fully halogenated compounds are already completely reduced.

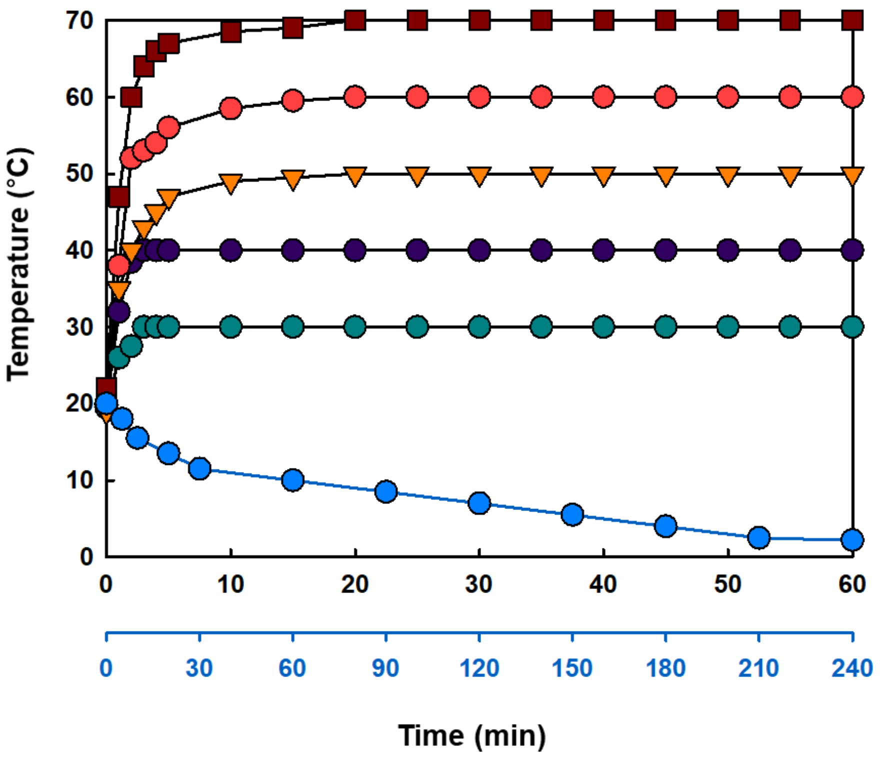

Figure S1. Temperature versus time profiles in the water bath and refrigerator. The water bath temperatures were set to 30 to 70°C and refrigerator was set to 2°C. The blue symbols and x axis offset indicate the time profile of sample temperature decrease in the refrigerator.

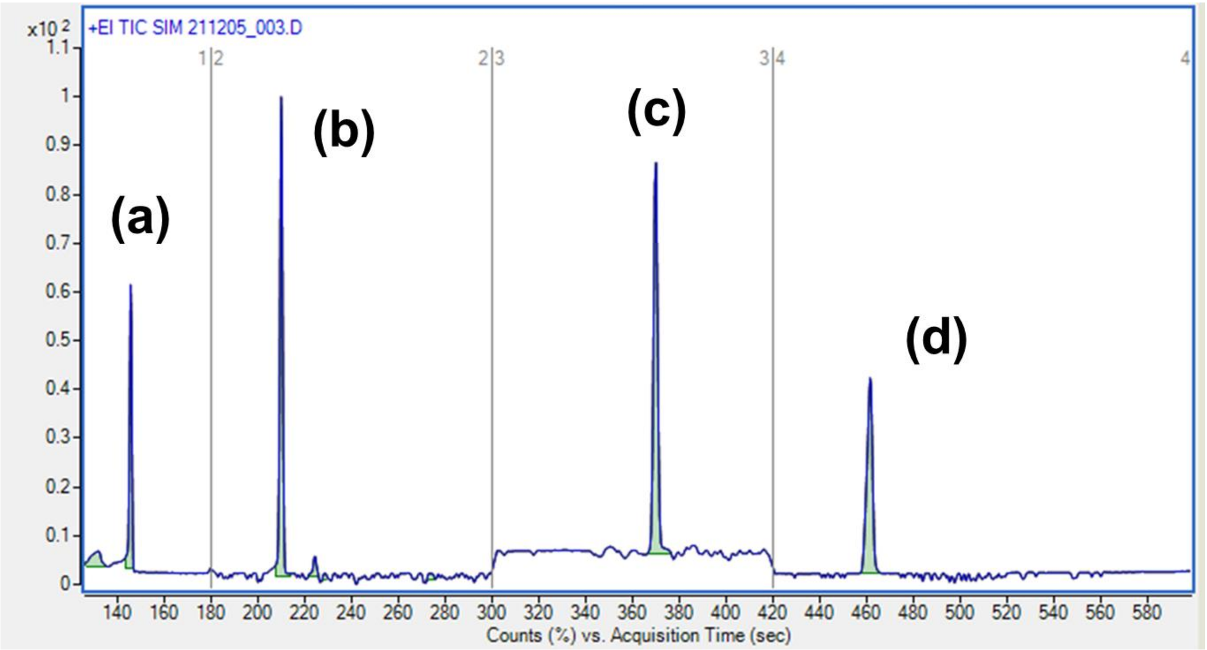

Figure S2. Representative GC/MS chromatogram of 0.76 μM trichloroethene (a), 0.6 μM tetrachloroethene (b), 0.49 μM pentachloroethane (c), and 0.42 μM hexachloroethane (d).

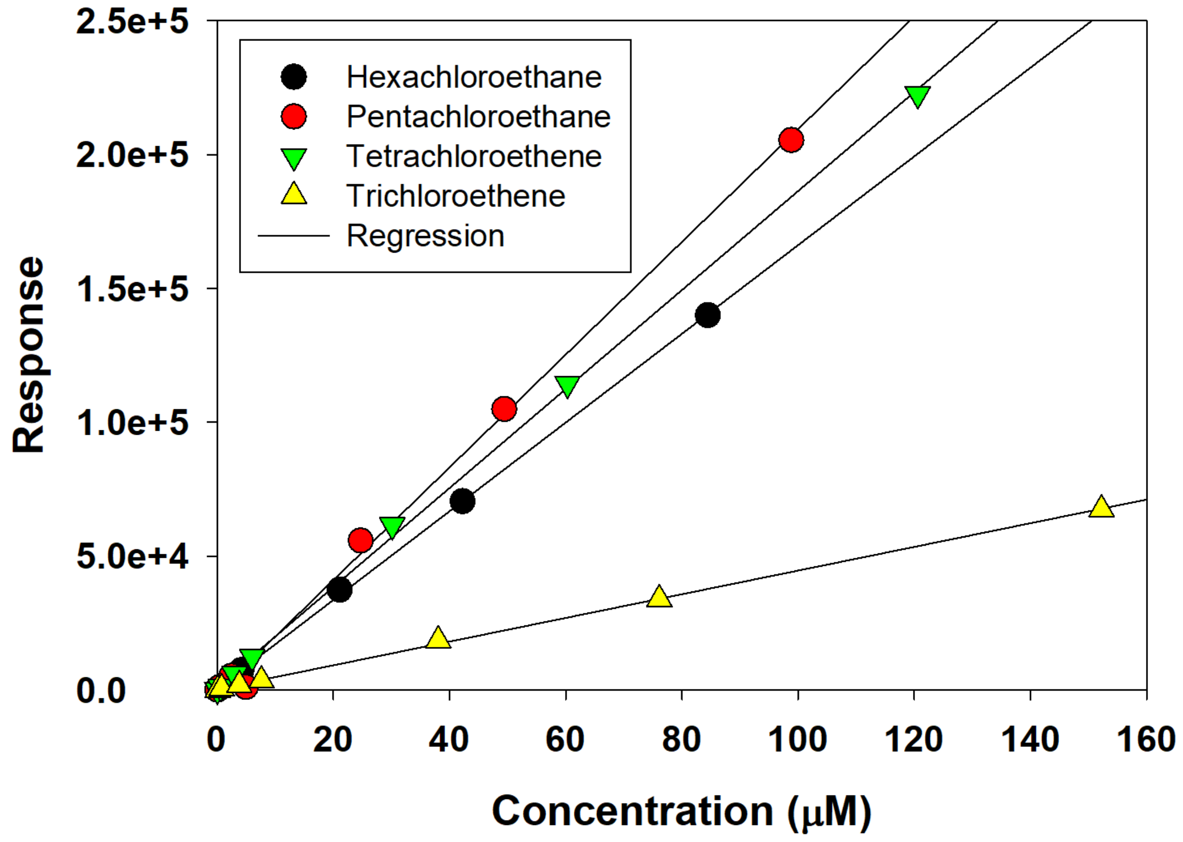

Figure S3. Target halogenated compound calibration curves.

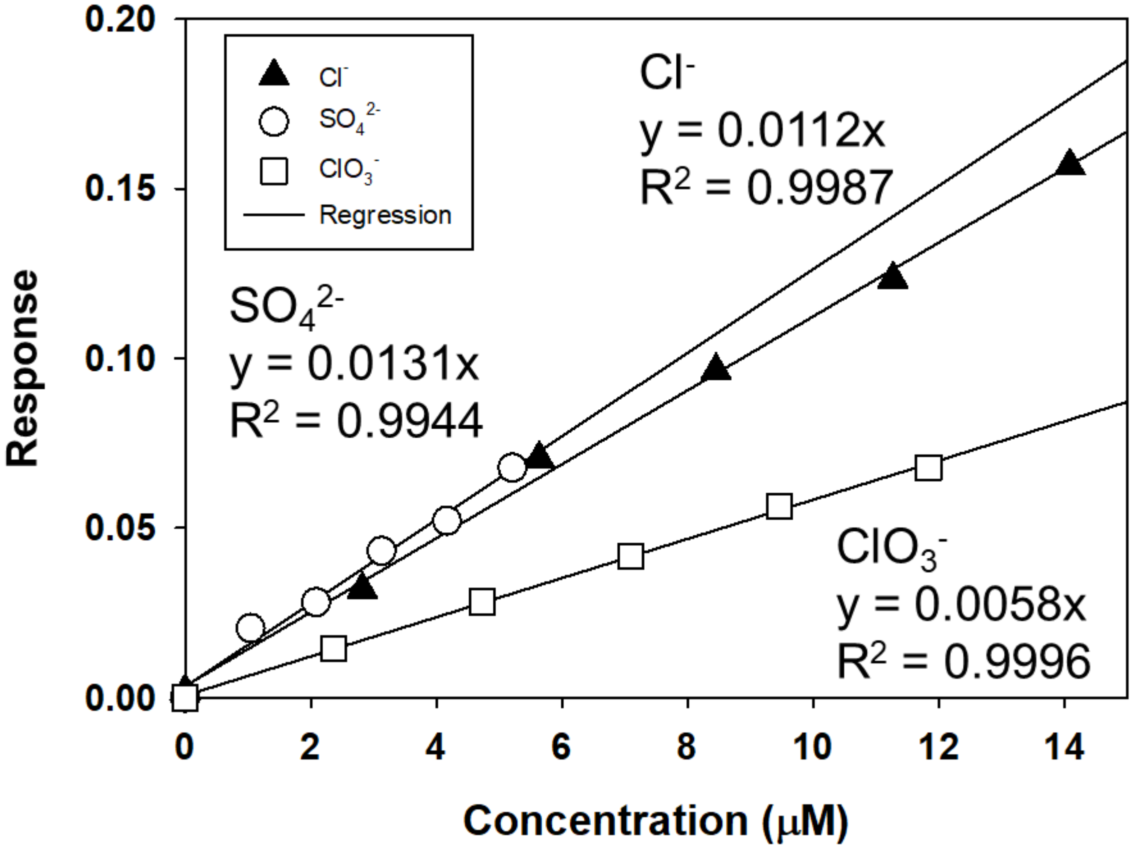

Figure S4. Representative anion calibration curves.

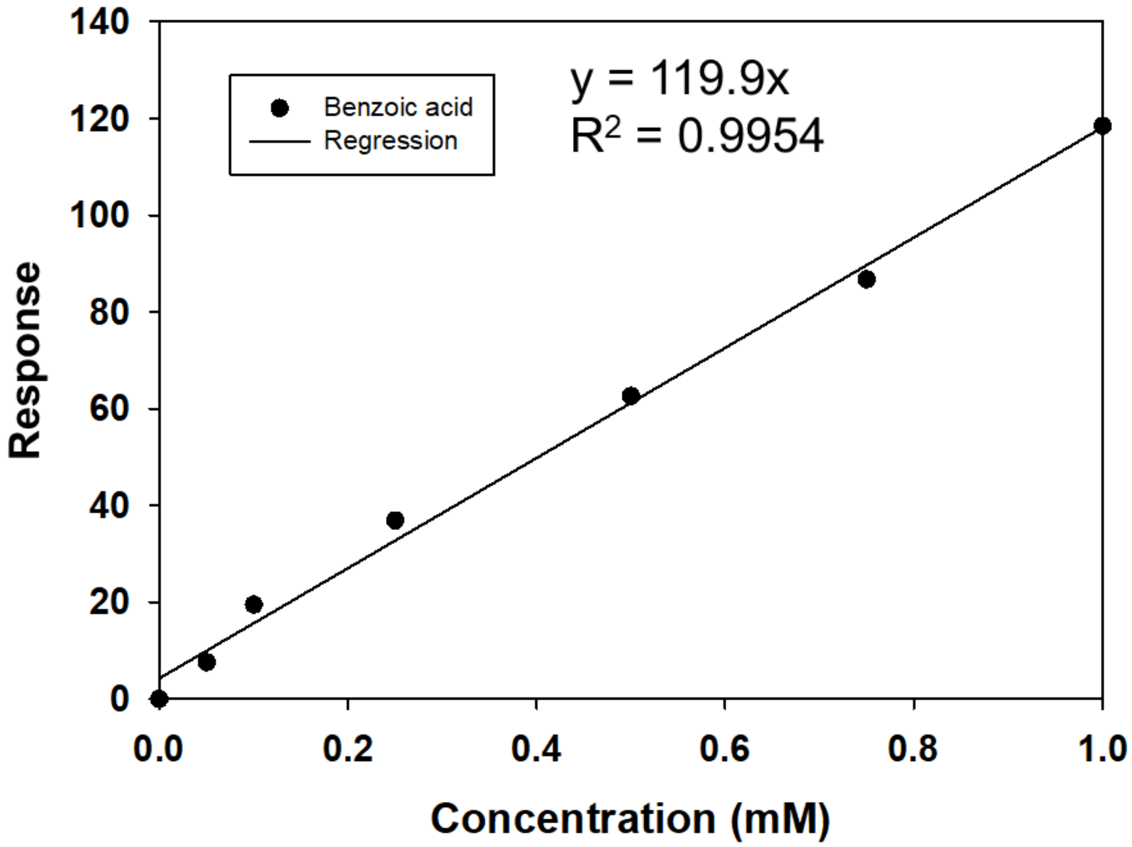

Figure S5. Representative benzoic acid calibration curves.

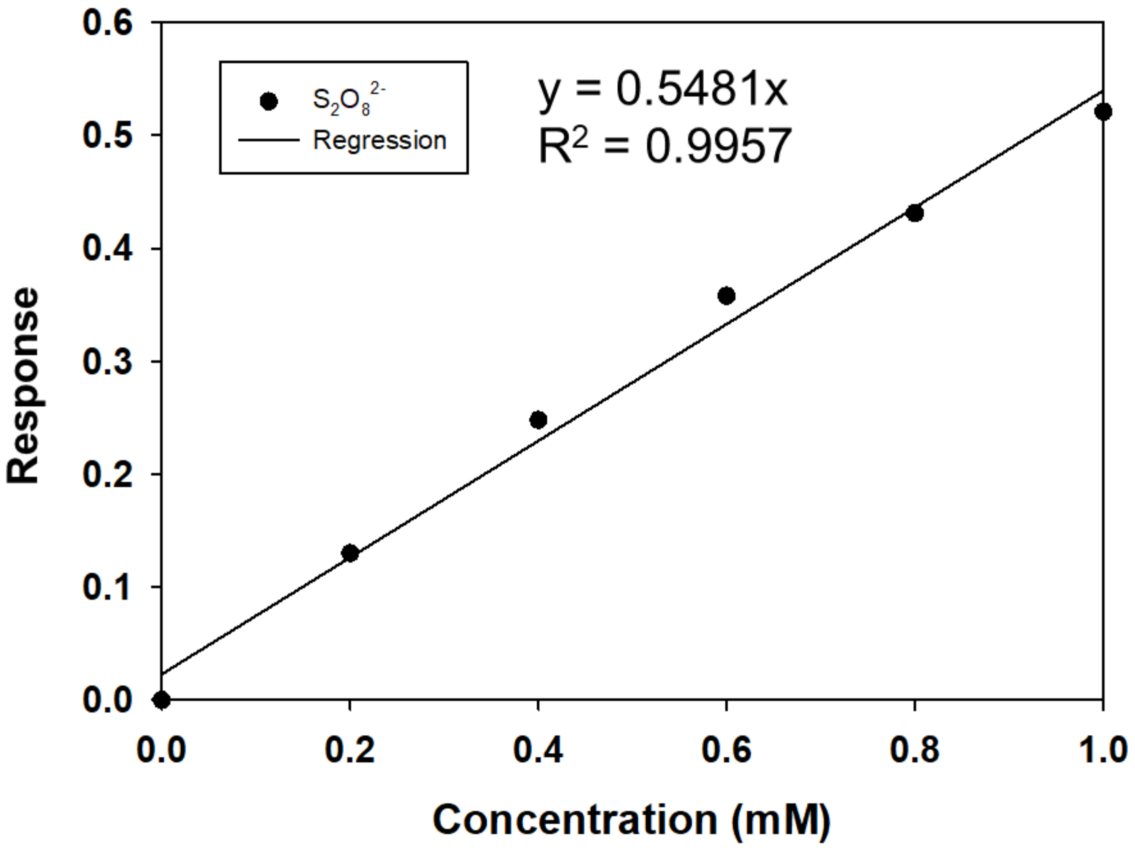

Figure S6. Representative $S_2O_8^{2-}$ calibration curves.

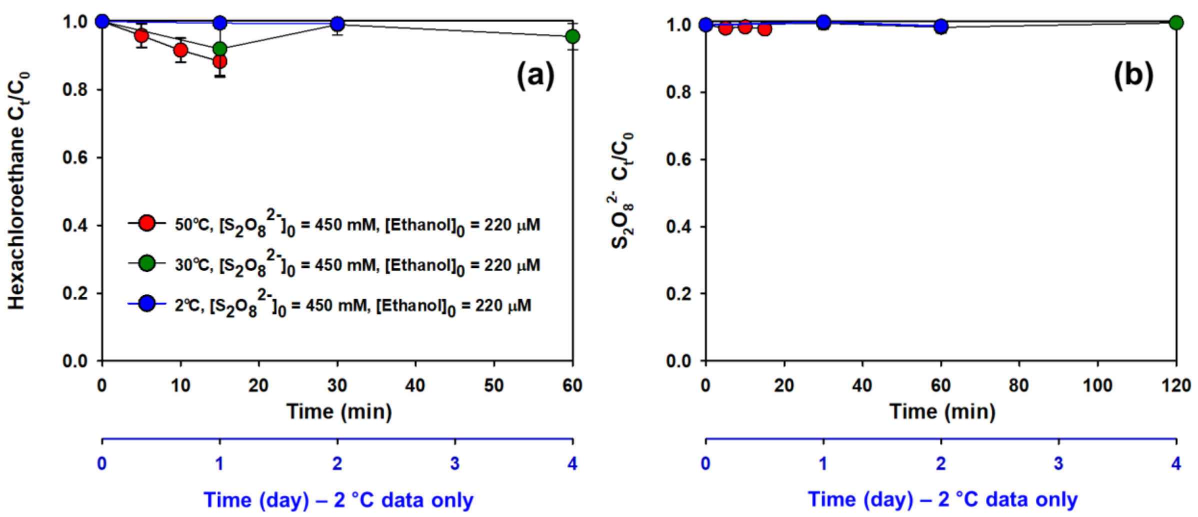

Figure S7. Transformation of hexachloroethane (a) and $S_2O_8^{2-}$ decomposition (b) in the presence of low concentrations of ethanol. X axis offset (blue color – day scale) indicates the time scale of 2°C data. 30°C and 50°C data refer to X axis (black color – min scale) ($[Ethanol]_0$ = 175 μM, $[O_2]_0$ = 220 to 275 μM, $[S_2O_8^{2-}]_0$ = 450 mM, $[Hexachloroethane]_0$ = 50 μM, $[Cl^-]_0$ = 1 mM, $[NO_3^-]_0$ = 0.1 mM, $[NO_2^-]_0$ = 0.01 mM, T = 2 to 50°C, pH_0 = 1.4).

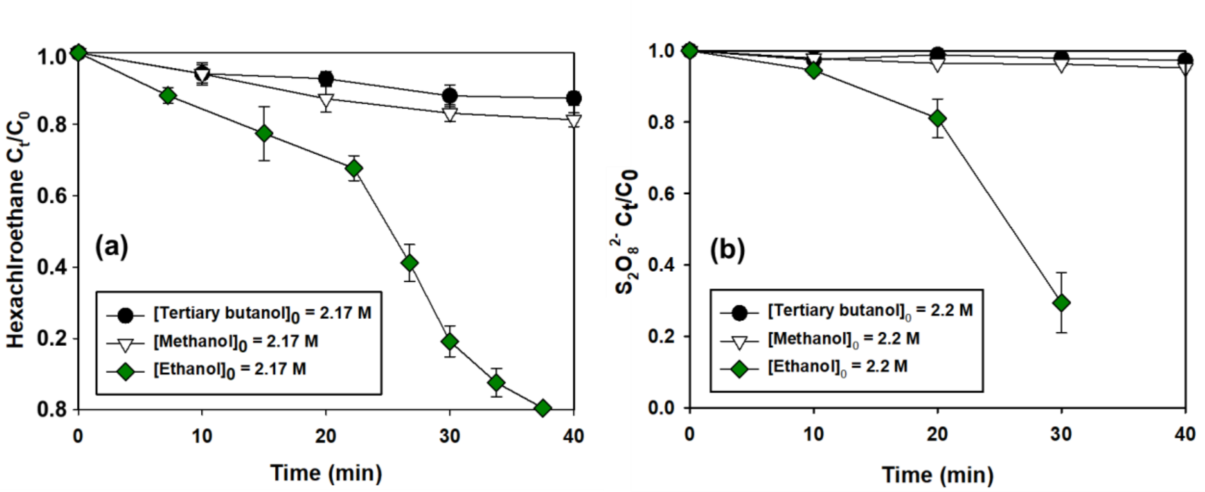

Figure S8. Hexachloroethane transformation (a) and decomposition of $S_2O_8^{2-}$ during heat-activated persulfate in the presence various alcohols (b) ([Tertiary butanol]$_0$ = 2.2 M, [Methanol]$_0$ = 2.2 M, [Ethanol]$_0$ = 1.8 M, [O_2]$_0$ = 150 μM, [$S_2O_8^{2-}$]$_0$ = 10 mM, [Hexachloroethane]$_0$ = 50 μM, Temp = 50°C, pH$_0$ = 4.3).

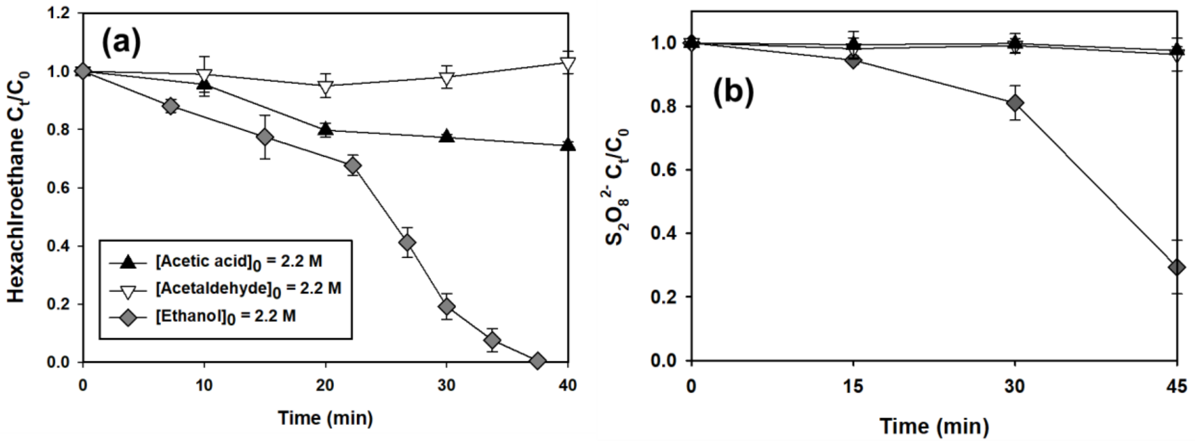

Figure S9. Transformation of hexachloroethane (a) and $S_2O_8^{2-}$ decomposition in the presence of ethanol, acetaldehyde and acetic acid ([Acetic acid]$_0$ = 2.2 M, [Acetaldehyde]$_0$ = 2.2 M, [Ethanol]$_0$ = 1.8 M, [O_2]$_0$ = 150 μM, [$S_2O_8^{2-}$]$_0$ = 10 mM, [Hexachloroethane]$_0$ = 50 μM, Temp = 50°C, pH$_0$ = 4.3).

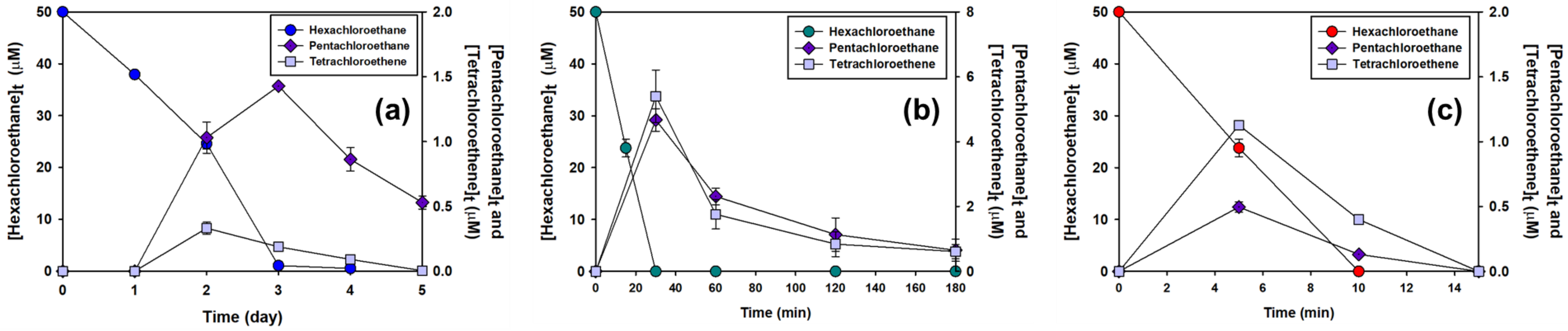

Figure S10. Enlarged version of hexachloroethane transformation and formation of dehalogenated products at 2°C (a); 30°C (b); and 50°C (c). ($[Ethanol]_0$ = 1.8 M, $[O_2]_0$ = 275 μM, $[S_2O_8^{2-}]_0$ = 450 mM, $[Cl^-]_0$ = 1 mM, $[NO_3^-]_0$ = 0.1 mM, $[NO_2^-]_0$ = 0.01 mM, pH_0 = 1.4).

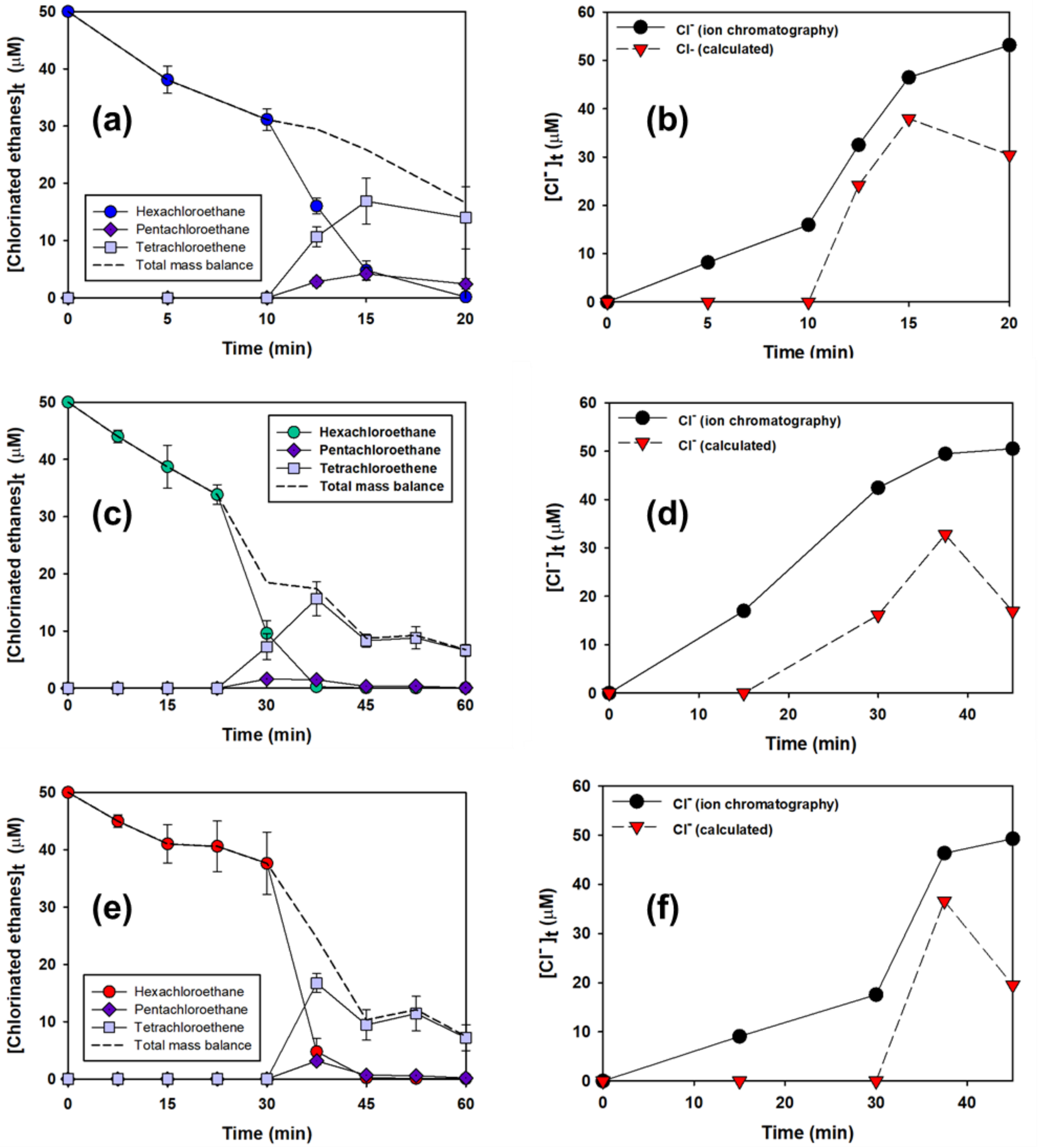

Figure S11. Loss of hexachloroethane and formation of dehalogenated products during decomposition of persulfate in the presence of ethanol at initial O_2 concentration ($[O_2]_0$) of 66 µM (a), 150 µM (c), and 225 µM (e). Release of chloride and estimated Cl^- contribution from measured dehalogenated products at $[O_2]_0$ of 66 µM (b), 150 µM (d), and 225 µM (f). Conditions: $[\text{Ethanol}]_0$ = 1.8 M, $[S_2O_8^{2-}]_0$ = 10 mM, $[\text{Hexachloroethane}]_0$ = 50 µM, Temp =

50°C, pH_0 = 4.3. Total mass balance is a sum of $[hexachloroethane]_t$, $[pentachloroethane]_t$, and $[tetrachloroethene]_t$. Calculated Cl^- can be determined by using following equation: Cl^- calculated = $[pentachloroethane]_t + 2[tetrachloroethene]_t$.

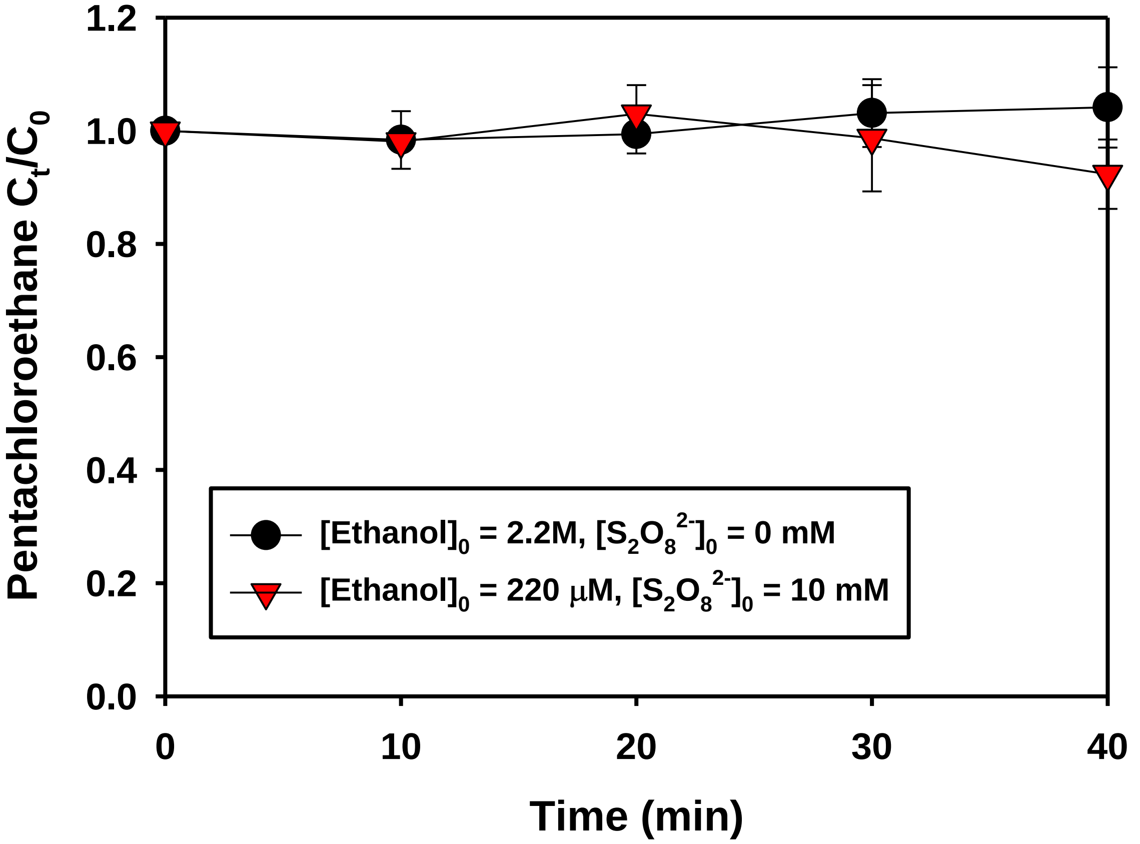

Figure S12. Transformation of pentachloroethane in the presence of ethanol without $S_2O_8^{2-}$ and in the presence of $S_2O_8^{2-}$ with 175 uM of ethanol. When persulfate was not added, a few drops of concentrated H_2SO_4 was used to adjust the initial pH value. To prevent oxidation of pnetachloroethane via $SO_4^{\bullet-}$ attack, 1 mM of benzoic acid was applied with $S_2O_8^{2-}$. Solutions were purged by N_2 to remove dissolved O_2. ([Ethanol]$_0$ = 175 μM and 1.8 M, [O_2]$_0$ = 70 μM, [$S_2O_8^{2-}$]$_0$ = 0 and 10 mM, [benzoic acid]$_0$ = 1 mM, [pentachloroethane]$_0$ = 50 μM, Temp = 50°C, pH_0 = 4.3).

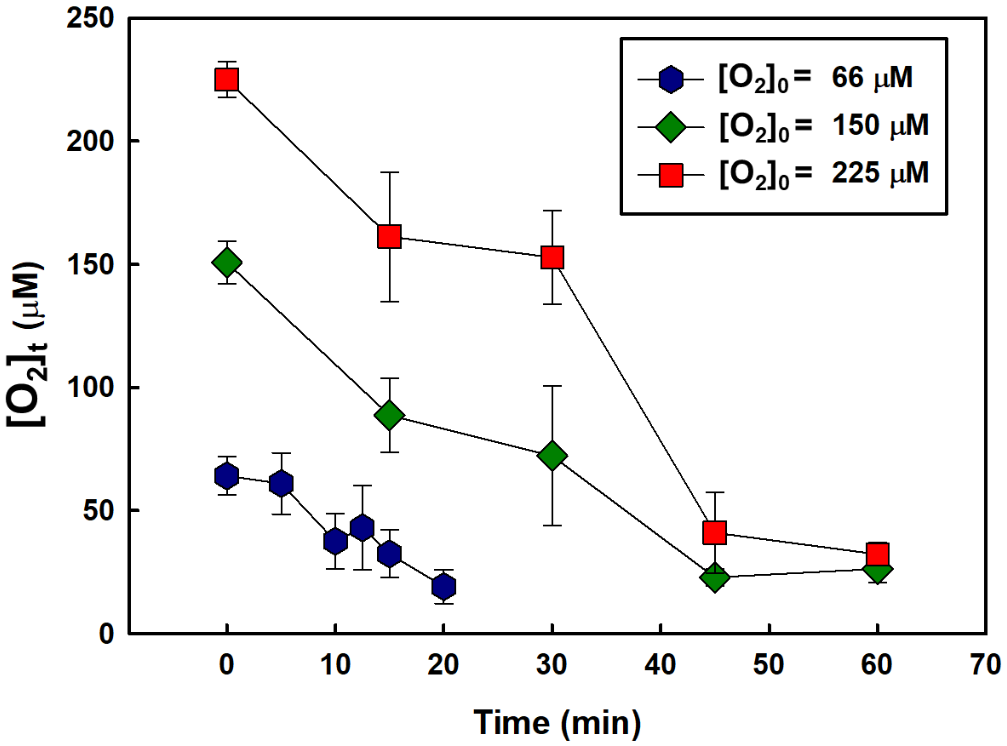

Figure S13. $[O_2]$ time profile during hexachloroethane transformation ($[O_2]_0$ = 66 to 225 μM, $[Ethanol]_0$ = 1.8 M, $[S_2O_8^{2-}]_0$ = 10 mM, [Hexachloroethane] = 50 μM, Temp = 50°C, pH_0 = 4.3).

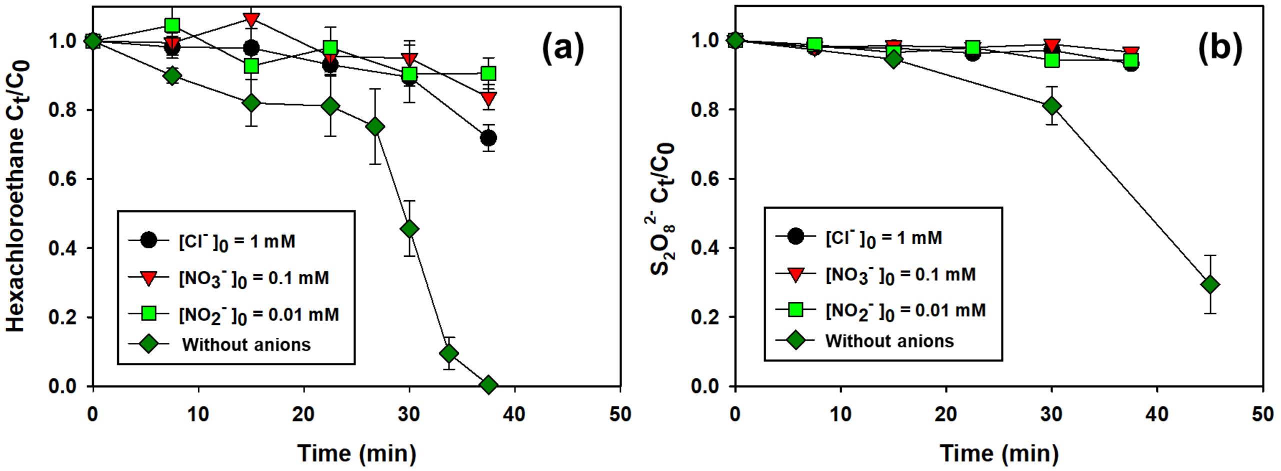

Figure S14. Effects of anions on hexachloroethane transformation (a) and $S_2O_8^{2-}$ consumption (b) under the low persulfate conditions ($[Ethanol]_0$ = 1.8 M, $[O_2]_0$ = 275 μM, $[S_2O_8^{2-}]_0$ = 10 mM, [Hexachloroethane] = 50 μM, $[Cl^-]_0$ = 1 mM, $[NO_3^-]_0$ = 0.1 mM, $[NO_2^-]_0$ = 0.01 mM, Temp = 50°C, pH_0 = 4.3).

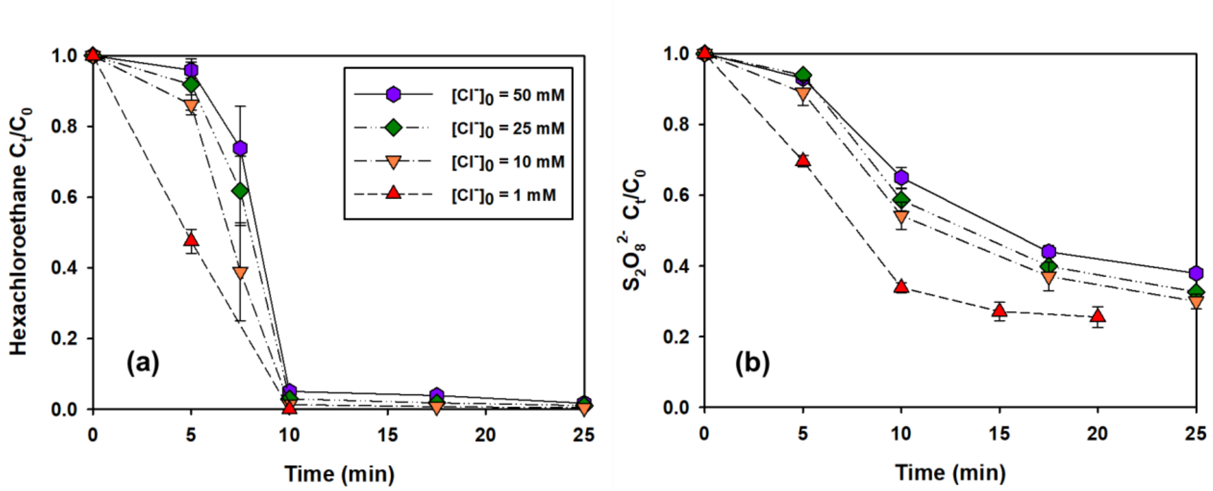

Figure S15. Effect of initial Cl^- concentrations on hexachloroethane transformation (a) and $S_2O_8^{2-}$ consumption (b) under the realistic ISCO conditions ([Ethanol]$_0$ = 1.8 M, $[O_2]_0$ = 275 μM, $[S_2O_8^{2-}]_0$ = 450 mM, [Hexachloroethane] = 50 μM, $[Cl^-]_0$ = 1 mM, $[NO_3^-]_0$ = 0.1 mM, $[NO_2^-]_0$ = 0.01 mM, Temp = 50°C, pH_0 = 1.4).

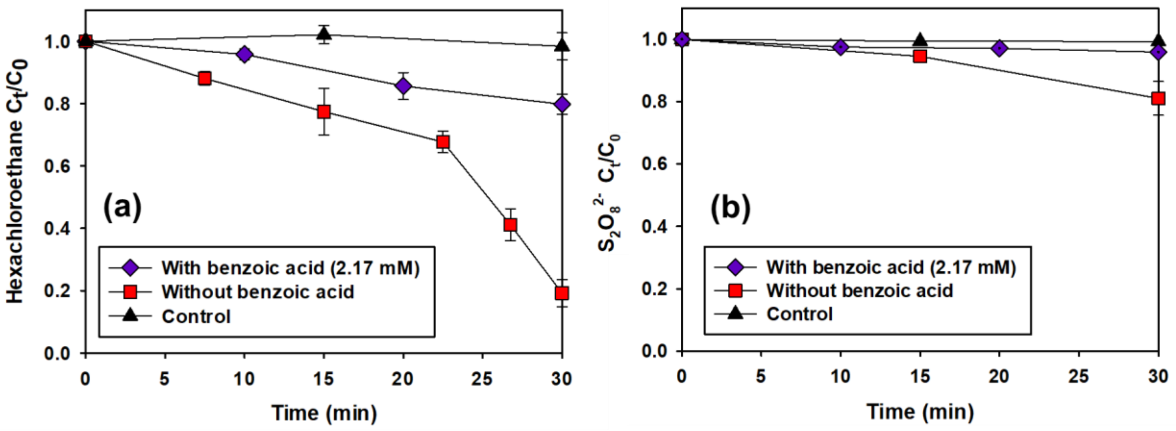

Figure S16. Effects of benzoic acid on hexachloroethane transformation (a) and $S_2O_8^{2-}$ consumption (b). Control experiments were conducted in the presence of 175 μM ethanol. ($[O_2]_0$ = 150 μM, $[\text{Ethanol}]_0$ = 175 μM and 1.8 M, $[S_2O_8^{2-}]_0$ = 10 mM, $[\text{Benzoic acid}]_0$ = 2 mM, $[\text{Hexachloroethane}]_0$ = 50 μM, Temp = 50°C, pH_0 = 4.3).

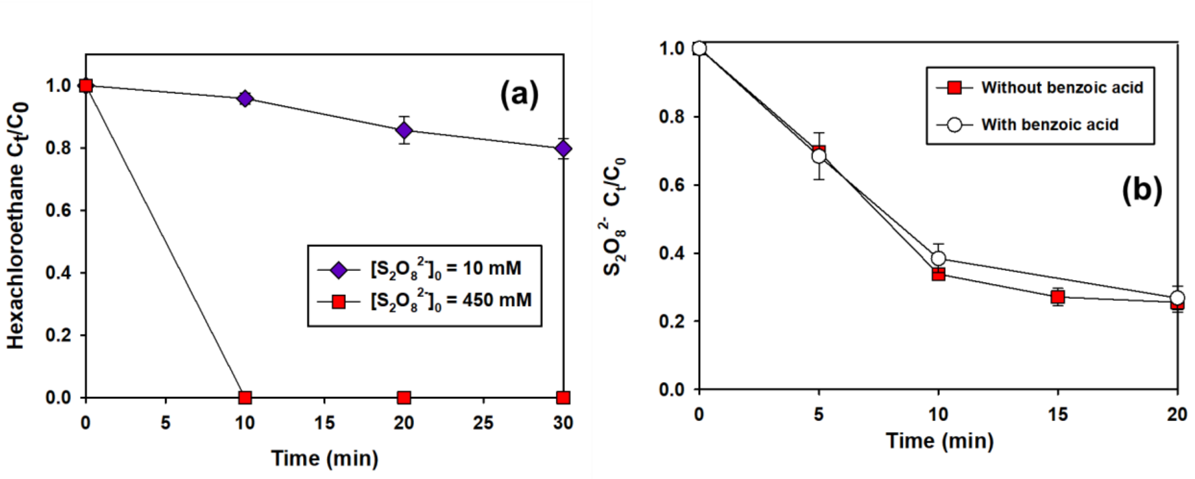

Figure S17. Effects of benzoic acid on hexachloroethane transformation in the presence of high or low concentrations of $S_2O_8^{2-}$ (a). $S_2O_8^{2-}$ consumption ($[S_2O_8^{2-}]_0$ = 450 mM) in the presence and absence of benzoic acid (b). When 10 mM of $S_2O_8^{2-}$ was applied, anions were not added. ($[Ethanol]_0$ = 1.8 M, $[O_2]_0$ = 255 µM, $[S_2O_8^{2-}]_0$ = 10 and 450 mM, $[Hexachloroethane]_0$ = 50 µM, $[Benzoic\ acid]_0$ = 2mM, $[Cl^-]_0$ = 0 and 1 mM, $[NO_3^-]_0$ = 0 and 0.1 mM, $[NO_2^-]_0$ = 0 and 0.01 mM, T = 50°C, pH_0 = 1.4 when 450 mM of $S_2O_8^2$- was applied, pH_0 = 4.3 when 10 mM of $S_2O_8^2$- was applied).

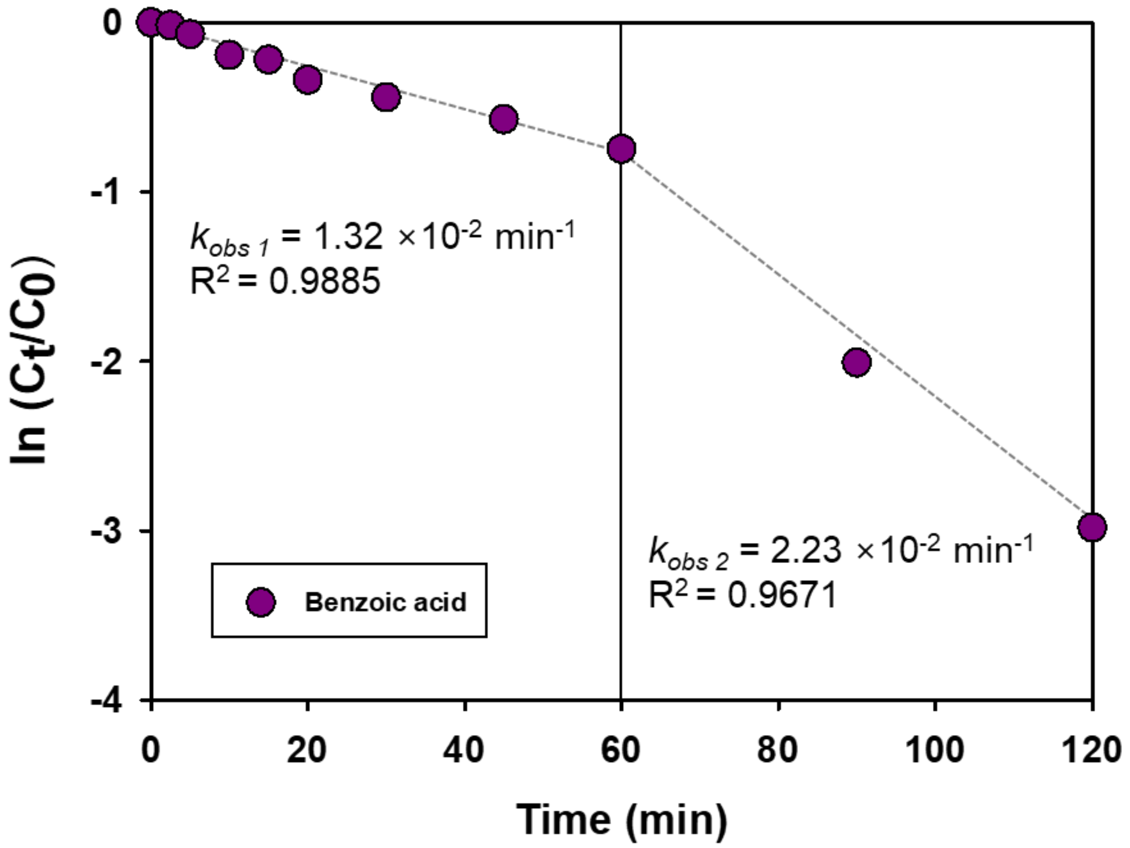

Figure S18. Degradation kinetics of benzoic acid during the first and second phases of the sequential treatment process ([Ethanol]$_0$ = 175 mM, [Benzoic acid] = 2 mM, [O_2]$_0$ = 280 μM, [$S_2O_8^{2-}$]$_0$ = 990 mM, Temp = 50°C, pH$_0$ = 1.4, n = 2).

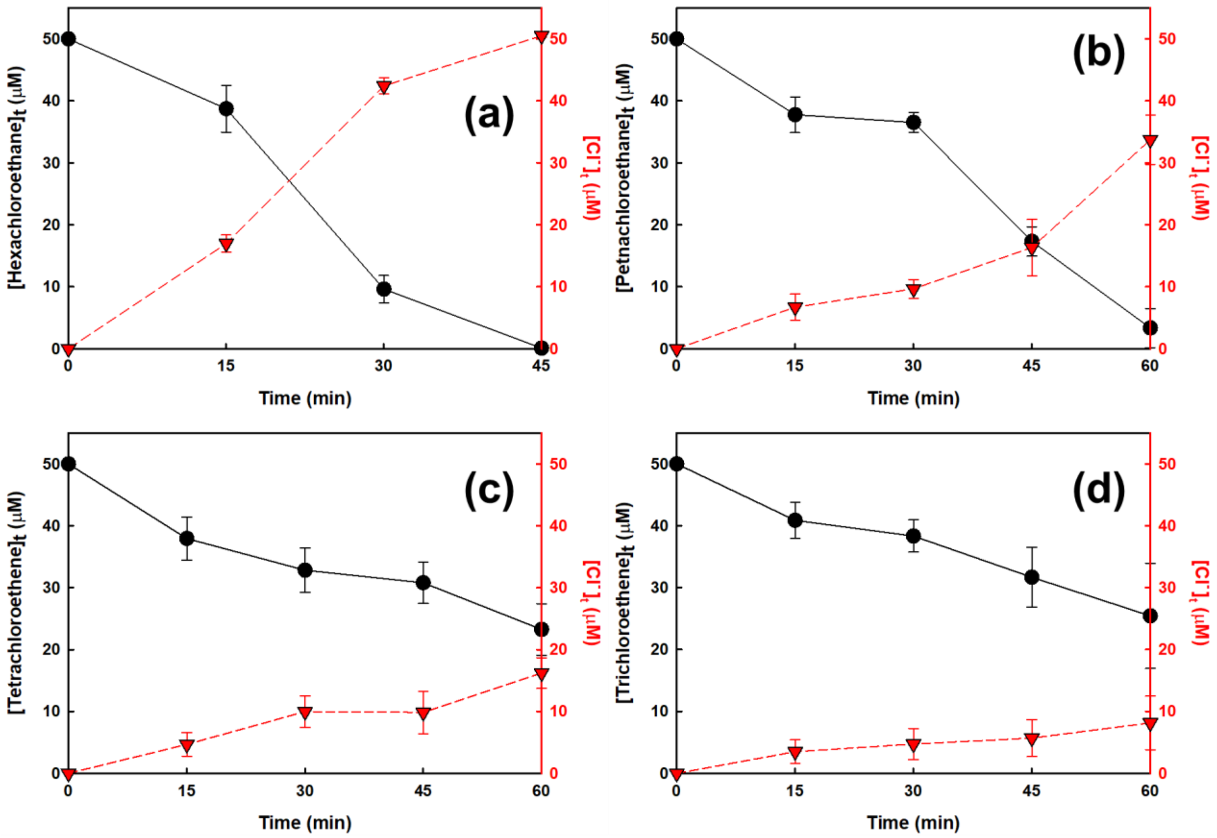

Figure S19. Transformation of hexachloroethane (a); pentachloroethane (b); tetrachloroethene (c); and trichloroethene (d) and recovery of Cl^- ($[\text{Hexachloroethane}]_0$ = 50 μM, $[\text{Pentachloroethane}]_0$ = 50 μM, $[\text{Tetrachloroethene}]_0$ = 50 μM, $[\text{Trichloroethene}]_0$ = 50 μM, $[O_2]_0$ = 150 μM, $[\text{Ethanol}]_0$ = 1.8 M, $[S_2O_8^{2-}]_0$ = 10 mM, T = 50°C, pH_0 = 4.2 to 4.5).

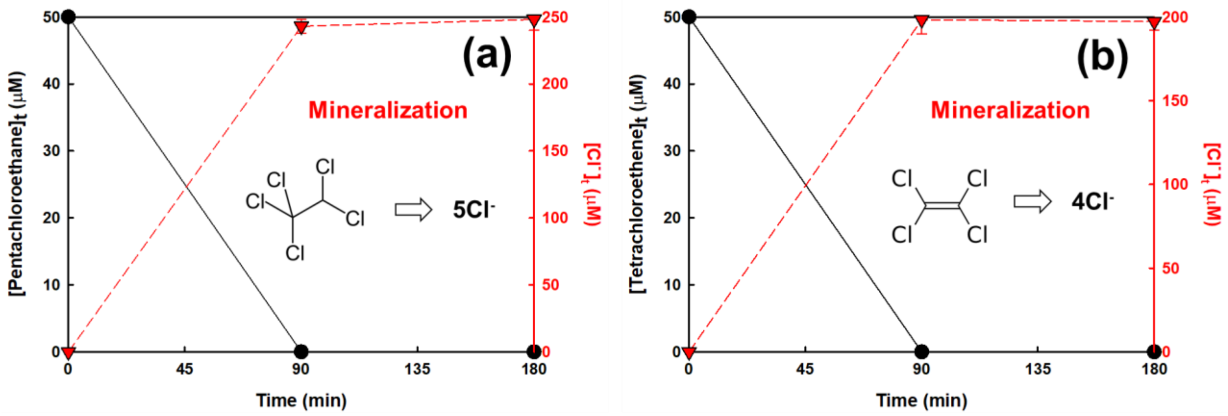

Figure S20. Pentachloroethane and tetrachloroethene mineralization during simulated second phase (oxidation) of process ($[O_2]_0$ = 225 μM, $[\text{Ethanol}]_0$ = 175 μM, $[S_2O_8^{2}]_0$ = 10 mM, $[\text{Pentachloroethane}]_0$ = 50 μM, $[\text{Tetrachloroethene}]_0$ = 50 μM, Temp = 50°C, pH_0 = 4.8). When persulfate was not added, a few drops of concentrated H_2SO_4 was used to adjust the initial pH value. Because chlorinated compounds are volatile, chloride analysis was conducted instead of using DOC to confirm the mineralization of each compound.

References

(1) Beylerian, N.; Khachatrian, A. The Mechanism of The Oxidation of Alcohols and Aldehydes with Peroxydisulphate Ion. *J. Chem. Soc., Perkin trans. II* **1984**, (12), 1937-1941.
